# Virus Propagation Linked to Exceedingly Rare Gene-Expression Errors: A Single-Molecule Microscopy Demonstration

**DOI:** 10.1021/acschembio.5c00638

**Published:** 2025-10-10

**Authors:** Raquel Luzón-Hidalgo, Gianluca D’Agostino, Valeria A. Risso, Asuncion Delgado, Beatriz Ibarra-Molero, Luis A. Campos, Jose Requejo-Isidro, Jose M. Sanchez-Ruiz

**Affiliations:** 1 Departamento de Quimica Fisica. Facultad de Ciencias, Unidad de Excelencia de Quimica Aplicada a Biomedicina y Medioambiente (UEQ), 16741 Universidad de Granada, Granada 18071, Spain; 2 Centro Nacional de Biotecnología (CNB), CSIC, Madrid 28049, Spain; 3 IMDEA-Nanociencia, Ciudad Universitaria de Cantoblanco, Madrid 28049, Spain; 4 Unidad de Nanobiotecnología, CNB-CSIC-IMDEA Nanociencia Associated Unit, Madrid 28049, Spain

## Abstract

Many viruses use programmed frameshifting and stop-codon misreading to synthesize functional proteins at high levels. The underlying mechanisms involve complex RNA sequence/structure motifs and likely reflect optimization driven by natural selection of inefficient, nonprogrammed processes. Then, it follows from basic evolutionary theory that low levels of proteins generated through gene expression errors could provide viruses with some survival advantage. Here, we devise an experimental demonstration of this possibility. Phage T7 recruits the host thioredoxin as an essential processivity factor for the viral DNA polymerase. We inserted early stop codons in the thioredoxin gene and appended to its end the sequence encoding for a photoconvertible fluorescent protein. Virus replication was not abolished. Single-molecule localization microscopy showed that the phage replicates even when there are only about 10 thioredoxin molecules per host cell on average, a number orders of magnitude below typical cellular protein levels. We show that this seemingly shocking result can be understood in molecular and evolutionary terms as a consequence of the polymerase-thioredoxin complex displaying high kinetic stability and a long residence time, as these are required to ensure high polymerase processivity. More generally, our demonstration that virus replication may be enabled by proteins at exceedingly low copy number suggests that viruses have access to the wide diversity of protein variants harboring phenotypic mutations as a result of gene expression errors. This mechanism could play a role, for instance, in cross-species transmission by enabling virus survival in the new host before adaptations appear at the genetic level.

## Graphical abstract

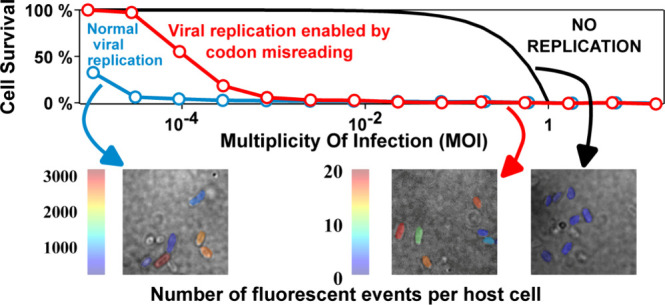

## Introduction

Viruses use programmed gene expression errors (i.e., recoding) to generate functional proteins. Specifically, viruses often use stop-codon misreading (i.e., stop-codon readthrough) and ribosomal frameshifting to synthesize two polypeptide chains from the same mRNA.^1–3^ These processes are programmed in the sense that sequence and structural motifs in the mRNA molecule guarantee that the resulting viral proteins are synthesized at comparatively high levels.^1–3^ However, the complex molecular mechanisms behind programmed gene expression errors cannot have emerged fully optimized in a single evolutionary event. Rather, they are necessarily the outcome of evolutionary processes that started with very low protein levels generated through inefficient, nonprogrammed processes. Yet, evolution has no foresight^4^ and a feature can only be enhanced by natural selection if it already provides a selective advantage to the organism. Basic evolutionary theory suggests, therefore, that the initial, low protein levels must have conferred the virus with a significant survival advantage, thus enabling the mutation-selection process of Darwinian evolution to act and promote the gene-expression error through RNA sequence modifications.

Here, we devise an experimental demonstration that extremely low protein levels arising from exceedingly rare gene expression errors may enable virus replication. Our demonstration targets a virus-host biomolecular interaction that is essential for the replication of a virus. We modify the gene of the host protein involved in the interaction (i.e., the host factor) in two ways. First, we insert early stop-codons, in such a way that the amount of host factor in the cell is reduced to the levels allowed by misreading of the stop-codon.^5^ Second, we append the sequence of a green/yellow photoconvertible fluorescent protein^6^ to the end of the host-factor gene corresponding to the C-terminus of the host factor, in such a way that even exceedingly low host-factor levels can be quantified. This is so because host factor molecules generated through codon misreading will carry an attached fluorescent probe that enables their detection through single-molecule localization microscopy^7^ (see graphical abstract).

In keeping with a long-established tradition of using bacteriophages to explore fundamental hypothesis in virology^8^ we applied the approach described above to the replication of phage T7 in its *E. coli* host, which requires the recruitment of the host thioredoxin as an essential processivity factor for the viral DNA polymerase.^9^ We took advantage of the availability of an *E. coli* knockout strain in which thioredoxin genes have been deleted.^10^ Since the essential host factor is missing, the phage cannot replicate in this knockout strain. Yet, viral replication is restored upon complementation of the knockout with a plasmid bearing the thioredoxin gene, even when an early, very-low-readthrough stop codon had been inserted, indicating that replication is enabled by the host factor at very low copy number. This was qualitatively supported by flow cytometry experiments and confirmed by single-molecule localization microscopy experiments which detected only about 10 thioredoxin molecules per host cell on average, a number many orders of magnitude below the typical cellular protein levels^11^ and at least 3 orders of magnitude below normal thioredoxin expression levels in *E. coli*.^12,13^

On an immediate level, our work sheds light on the biophysical features that may shape the evolution of virus-host biomolecular interactions. The fact that the virus replicates even after a drop of several orders of magnitude in thioredoxin level, immediately indicates that the extremely tight (nanomolar)^14,15^ polymerase-thioredoxin interaction cannot solely reflect natural selection for a favorable binding thermodynamics. An explanation consistent with our experimental results can, however, be proposed on the basis of the known biological function of the host factor and the related kinetic stability requirements. Briefly (see Discussion for details) the very tight, nanomolar interaction likely reflects natural selection for a long residence time of the thioredoxin-polymerase complex (i.e., for a low value of the dissociation rate constant) as it is required to ensure high polymerase processivity.

More generally, our work points to the possibility that viruses have access to the wide diversity of protein variants generated through gene expression errors. This notion is not unreasonable since viruses can achieve huge amplifications on the basis of a few crucial biomolecular interactions in a few host cells,^17^ and it is specifically supported by our experimental demonstration that phage T7 replication is enabled by a host factor at very low copy number. Many gene expression errors lead to amino acid replacements, i.e., to the so-called phenotypic mutations, which are orders of magnitude more frequent than genetic mutations.^18–21^ According to recent estimates,^21^ about 20% of protein molecules bear phenotypic mutations, indicating that each given protein species is an ensemble of many different molecules, including a major contribution from the wild-type form but also including a huge diversity of variants, each at low level. Therefore, it appears possible that, in some scenarios, biomolecular interactions required for virus replication are enabled by a few molecules bearing suitable phenotypic mutations (see Discussion for details). As an illustrative example, we discuss that this mechanism could play a role in cross-species transmission by allowing the virus to initially survive in the alien molecular environment of the new host, thus giving natural selection a chance to act and generate the required adaptations at the genetic level.

## Results

### Virus, Host Strain and Host Factor

Bacteriophage T7 is a lytic phage that infects strains of *E. coli* and destroys the infected cells, with the concomitant release of more than a hundred of new virions per infected cell.^22^ Infection starts with the attachment of a virion to the cell surface followed by transfer of the viral DNA to the cytosol. This triggers processes that eventually lead to the death of the cell. In addition to infection, phage T7 propagation requires, among other processes, the assembly inside the host cell of a viral replication machinery, in such a way that copies of the viral DNA are generated to be subsequently packed inside the new virions. The minimalist replisome of bacteriophage T7 consists of a DNA polymerase, a helicase/primase, a ssDNA-binding protein and a host thioredoxin.^15^

Thioredoxins are small redox proteins involved in a diversity of processes in all known cells.^16,23^ Crucially, the thioredoxin in the replisome of phage T7 is a protein from the *E. coli* host that is recruited by the virus to serve, not as a redox enzyme, but as an essential processivity factor for the viral DNA polymerase (Figure 1A).^9^ *E. coli* thioredoxin (Figure 1B) is expressed in *E. coli* to copy numbers of about 10000–20000 molecules per cell.^20,21^ The immediate question we asked in this work is whether replisome assembly and subsequent replication of phage T7 requires the presence of ∼ 10^4^ thioredoxin molecules in a host cell or whether a much smaller number of thioredoxin molecules per cell suffices to trigger virus propagation.

**Figure 1:**
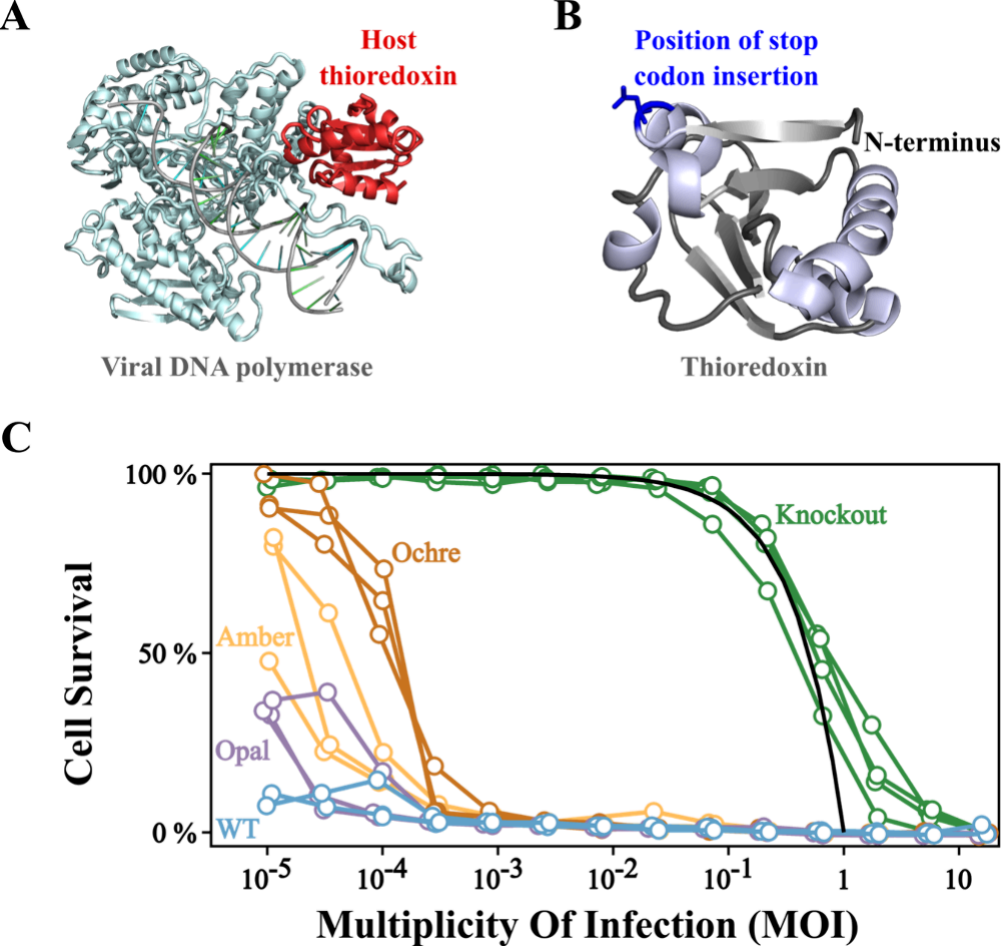
Host-factor engineering of phage T7 propagation. (A) Structure of the viral DNA polymerase of phage T7 interacting with the host thioredoxin (PDB ID 1T8E). Thioredoxin functions as an essential processivity factor for the polymerase.^9^ The interaction of the two proteins is tight with a reported dissociation constant of 5 nM.^15^ (B) 3D-structure of *E. coli* thioredoxin (PDB ID 2TRX) showing the position of insertion of stop codon (position 11). (C) Propagation of phage T7 in *E. coli* cells with modified expression of the host factor. Experiments were performed with knockout Trx^–^ cells, in which thioredoxin is absent, with cells in which the thioredoxin gene had been modified through the insertion of opal, amber and ochre stop codons, and with cells transformed with the unmodified (wt) thioredoxin gene. For each type of cell, propagation experiments with different initial multiplicity of infection (MOI) values were set up and the extent of cellular death was assessed by turbidimetry after 1 h (see Materials and Methods for details). For each type of cell, three propagation profiles (fraction of cell survival vs MOI) were determined using three biological replicates (4 biological replicates for the knockout cells). Phage does not replicate in knockout Trx^–^ cells, since they do not express the essential host factor, thioredoxin. Consequently, death of knockout cells requires MOI values approaching unity. In fact, the continuous line is the theoretical prediction if the fraction of cell death equals the MOI value. By contrast, complete death is observed for all the other types of cells, even with very low MOI values. An efficiency of propagation ranking of ochre < amber < opal < wt is visually apparent and likely reflects the generated thioredoxin levels.

To address the question posed above, we made use of a previously characterized^10^ knockout *E. coli* strain in which thioredoxin genes have been deleted. Specifically, this work uses the strain FA41, which is deficient in the two thioredoxins described in *E. coli*: thioredoxin 1 (which is referred to throughout this work simply as thioredoxin) and thioredoxin 2, which is induced under stress conditions and bears a Zn-binding domain. We shall refer to this strain as *E. coli* Trx^–^. This strain can grow, albeit more slowly than the corresponding wild-type DHB4 strain, likely because the glutaredoxin pathway substitutes to some extent the functions of thioredoxin. The knockout *E. coli* Trx^–^ effectively decouples virus infection from virus replication. That is, *E. coli* Trx^–^ cells are infected by the phage and die as a result of the infection, but they cannot amplify the virus, since the absence of thioredoxin precludes the assembly of efficient viral replisomes. Consequently, death of all the cells in a sample of *E. coli* Trx^–^ requires a large multiplicity of infection (MOI: the ratio of number of virus particles to number of host cells) of about unity or larger, as it is visually apparent in Figure 1C. By contrast, all cells in a wild-type *E. coli* sample are eventually killed by the phage, even in experiments with very low MOI values, simply because the synthesis of thioredoxin molecules enables replisome assembly and, consequently, initial infection of just a few cells generates many new virions that propagate the infection. In general, virus replication is revealed by the death of essentially all cells in propagation experiments that start with MOI values much smaller than unity.

### Stop-Codon Engineering Reveals Virus Replication Enabled by a Few Host Factor Molecules per Cell

We complemented the knockout *E. coli* Trx^–^ cells with a plasmid harboring the gene of *E. coli* thioredoxin, which allows us to easily modify thioredoxin in several useful ways.^10^ The basal expression of *trxA* from the promoter of the pET30a­(+)::*trxA* plasmid we used already produces thioredoxin levels similar to those corresponding to the constitutive expression in wild-type *E. coli* cells (ref 10 and results given further below). In fact, even under noninducing conditions, complementation restores the phage infection pattern of wild-type *E. coli*, i.e., essentially all cells eventually die in experiments starting with very low MOIs (Figure 1C). That is, the basal expression produces a thioredoxin level comparable to the constitutive expression in WT *E. coli* cells and enables virus propagation with an efficiency similar to that observed with WT cells, thus providing a useful and convenient reference. In view of this, we decided to decrease the expression levels by introducing early stop codons in the thioredoxin gene. It has been known for many years that stop codons are “leaky” to different extents as a result of, for instance, transcription errors or translational readthrough.^14^ Therefore, we hypothesized that early stop codons would not completely block thioredoxin synthesis but would lead to substantially decreased thioredoxin copy numbers, depending on the leakiness of the codon.

Position 11 was selected for stop-codon insertion because it is exposed to the solvent in the 3D-structure of thioredoxin (Figure 1B) and shows considerable diversity among thioredoxins from different species (supplementary Figure S1). The reason for choosing a position with high exposure to solvent in the native protein structure was to prevent as much as possible the possibility that an amino acid residue generated by misreading impaired proper thioredoxin folding, something that could easily happen if we had selected a buried position (at which the amino acid residue would be involved in interactions with other residues). That is, different amino acid residues at position 11 (result of the misreading of the stop-codon) are likely to lead to correctly folded thioredoxin molecules. We introduced opal, amber and ochre stop codons at position 11 and found clear evidence of virus replication in the three cases (Figure 1C and supplementary Figure S2). The number of plaque-forming units (PFU) for Trx^–^ cells transformed with opal-trx, amber-trx and ochre-trx genes were thus on the same order as the PFU values for cells transformed with wild type thioredoxin. However, propagation was less efficient when stop codons had been inserted. Furthermore, among the genes with stop codons inserted, propagation was least efficient with the ochre stop codon and most efficient with the opal stop codon. This is clearly shown by the profiles of cell survival versus MOI (Figure 1C) and by the size of the plaques (supplementary Figure S2) in experiments aimed at determining the number of PFU values. Remarkably, the observed ranking of propagation efficiency ochre < amber < opal (Figure 1C and supplementary Figure S2) matches the known ranking of stop-codon leakiness in *E. coli*. That is, opal is known to be particularly leaky while ochre shows the lowest readthrough frequency.^5^ The congruence supports that the differences in propagation efficiency reflect the number of generated thioredoxin molecules and consequently the number of assembled replisomes.

The fact that the presence of the ochre codon does not block replication already provides a first, qualitative estimate of the number of host factor molecules per cell required to enable virus replication. An early ochre stop codon is expected to reduce the expression levels by a factor corresponding to its readthrough frequency. Normal thioredoxin expression in *E. coli* leads to copy numbers of 10000–20000 molecules per cell^12,13^ and the readthrough frequency of the ochre codon has been estimated in the range 10^–5^ to 9·10^–4^.^5^ A straightforward calculation thus indicates that, upon insertion of an early ochre codon in the thioredoxin gene, the number of thioredoxin molecules synthesized per cell would drop to a value within the approximate range 1–20.

Phage replication enabled by about 20 or less host factor molecules is a rather shocking result. We, therefore, sought to obtain a direct and independent assessment of the number of thioredoxin molecules generated through ochre codon misreading. We made a first attempt at detecting the amount of thioredoxin synthesized in Trx^–^ cells transformed with ochre-trx genes in Western blots. However, no thioredoxin could be detected in these cells using this approach (supplementary Figure S3). This result should not come as a surprise since copy numbers of around 20 molecules per cell imply that the total amount of thioredoxin in the sample would be far below the levels typically detected in Western blots, which are typically on the order of tens of nanograms of the targeted protein.^24^ Actually, reaching the protein levels typically detected through Western Blotting for thioredoxin in Trx^–^ cells transformed with ochre-trx genes would have required using unrealistically large sample volumes (see legend to supplementary Figure S3 for details).

In view of the above, we decided to explore approaches based on the use of fluorescent probes, which may potentially detect molecules at low copy number. We started by appending to the end of the thioredoxin gene (Figure 2A) the sequence encoding for the enhanced green fluorescent protein (eGFP). In this way, every synthesized thioredoxin molecule is expected to carry an attached eGFP molecule and eGFP fluorescence could provide a metric of the amount of thioredoxin generated. As expected, knockout Trx^–^ cells transformed with wt-trx-eGFP, i.e., with the construct bearing the intact wild-type thioredoxin gene, show high fluorescence levels (Figure 2A), reflecting the normal thioredoxin expression levels. On the other hand, introduction of an ochre stop at position 11 of thioredoxin (Figure 2B) leads to fluorescence levels that are very low and similar, for almost all cells, to those observed with the knockout *E. coli* Trx^–^ cells (Figure 2C) that lack thioredoxin and the eGFP genes. Accordingly, the fluorescence levels of the cells transformed with the ochre-trx-eGFP construct are barely distinguishable from the autofluorescence levels. Autofluorescence in cells is a well-documented phenomenon that arises primarily from endogenous biomolecules containing aromatic amino acids. Key contributions include NAD­(P)­H, flavins and lipopigments.^25^ It is likely that, in our experiments, flavins provide the major contribution to autofluorescence, since they are efficiently excited at 488 nm and emit within the standard detection range for GFP.^25^ In any case, in ochre-trx-eGFP transformed cells, the contributions from endogenous autofluorescence and eGFP are comparable and in only a small fraction of these cells the fluorescence levels are (slightly) above autofluorescence indicating detectable, albeit very low, eGFP expression (supplementary Figure S4). As described below, this scenario was confirmed by the cell population distributions over fluorescence intensity in the emission window of eGFP derived from flow cytometry experiments (see Materials and Methods for details).

**Figure 2:**
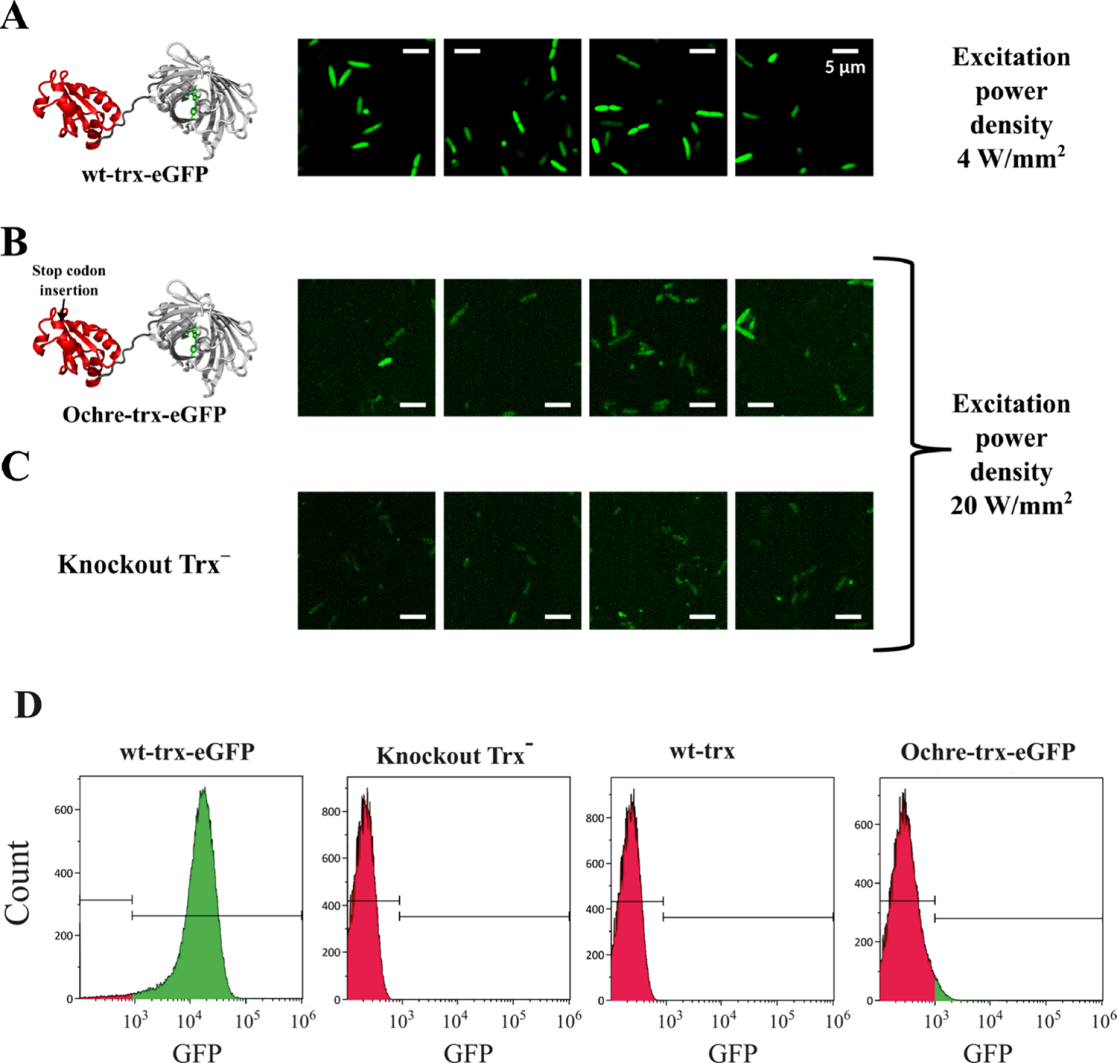
Assessment of thioredoxin levels using eGFP fusions. (A) Depiction of the 3D-structure of the wt-trx-eGFP fusion construct and four representative microscopy images of cells transformed with wt-trx-eGFP. (B) Depiction of the 3D-structure of the ochre-trx-eGFP fusion construct with the position of insertion of the ochre codon highlighted and four representative microscopy images of cells transformed with ochre-trx-eGFP. (C) Four representative microscopy images of knockout Trx^–^ cells. All images in panels (A-C) are displayed at the same spatial resolution shown in the rightmost top panel (scale bar: 5 μm). Similarly, all images have been rendered with the same contrast settings, although images in B–C were acquired at 5 times higher power density. See supplementary Figure S3 for further details. (D) Cell population distributions derived from flow cytometry in the emission window of eGFP. For each type of cell, three independent experiments were performed with very similar results. Here, we show one representative experiment for each type of cell, while all the experiments are collected in supplementary Figure S5. The fluorescence distributions for knockout Trx^–^ cells and those complemented with wild-type trx (without eGFP) reflect only cellular autofluorescence, with signal intensities remaining below 10^3^. In contrast, cells transformed with wt-trx-eGFP and ochre-trx-eGFP constructs exhibit detectable fluorescence above 10^3^ (highlighted in green). However, this fluorescence is substantially reduced in the ochre-trx-eGFP group: 97.58% of wt-trx-eGFP cells exceed the 10^3^ threshold, compared to only 8.22% in the ochre-trx-eGFP population. The structures of the fusion proteins (panels A and B) are shown for illustration only and were constructed by connecting through the linker the structures of the partners taken from the Protein Data bank. No attempt was made to refine the structure of the linker, since it is expected to be highly flexible.

As shown in Figure 2D, the fluorescence distributions for knockout *E. coli* Trx^–^ cells, as well as for knockout *E. coli* Trx^–^ cells complemented with a plasmid harboring the gene of *E. coli* thioredoxin (with no eGFP attached), are comparatively narrow and display a maximum at very low fluorescence intensity. These distributions obviously reflect cell autofluorescence, as they correspond to cells that do not express eGFP.^25^ By contrast, the distribution for the Trx^–^ cells transformed with wt-trx-eGFP is comparatively wide and shows a maximum at about 3 orders of magnitude above that of the knockout Trx^–^ cells, reflecting the expression of the wt-trx-eGFP construct. Remarkably, the distribution for ochre-trx-eGFP transformed cells is similar, but not totally identical, to that of cells that do not express eGFP (Figure 2D). That is, the distribution for ochre-trx-eGFP transformed cells also displays a maximum at low fluorescence intensity, as it is the case for cells that do not express eGFP, but the distribution is slightly wider with a distinct high-fluorescence tail (shown in green color in the corresponding panel in Figure 2D). Clearly, while the distribution for Trx^–^ cells transformed with ochre-trx-eGFP is dominated by autofluorescence, it also reveals a minor contribution due to the eGFP molecules expressed as a result of stop-codon misreading. This contribution overlaps to a substantial extent with that from autofluorescence, resulting on the whole in fluorescence levels about 3 orders of magnitude below those observed for cells expressing the wt-trx-eGFP construct.

Overall, the experiments shown in Figure 2 with the eGFP constructs support that the enabling thioredoxin levels generated through ochre-codon misreading are indeed exceedingly low. Yet, it does not appear possible to use eGFP fluorescence to quantify these levels. In view of this, we decided to explore a different approach based on appending to the thioredoxin gene the sequence encoding for the photoconvertible fluorescent protein mEos2 ^6^ and using single molecule fluorescence microscopy. These experiments are described in the next sections.

### Host-Factor Levels from Single-Molecule Localization Microscopy

We appended to the thioredoxin gene the sequence encoding for the photoconvertible fluorescent protein mEos2.^6^ In this way the synthesized thioredoxin molecules are linked to mEos2 (Figure 3A), as we ascertained from the UV–vis spectra of the purified fusion construct (Figure 3A). Crucially, the presence of mEos2 in the final protein product of thioredoxin gene expression allows us to use single-molecule localization microscopy (SMLM)^7^ to quantify the number of generated thioredoxin molecules. To set up the relevant methodological details, we first used a construct including wild type thioredoxin (wt-trx-mEos2). That is, we transformed the knockout Trx^–^ cells with the wt-trx-mEos2 gene. Since no stop codon is present, we expect normal expression levels of about 10000–20000 thioredoxin molecules per cell.^12,13^ Certainly, most of the thioredoxin molecules thus generated will bear an attached mEos2 and there could be some doubt as to whether the presence of the photoconvertible proteins prevents recruitment of the thioredoxin moiety by the viral DNA polymerase. However, virus propagation experiments show that the presence of the attached mEos2 does not preclude the death of all cells even when the experiments start with low MOI values down to ∼ 10^–4^ (Figure 3B; see also supplementary Figure S2) thus supporting recruitment and the subsequent processivity of the viral DNA polymerase.

**Figure 3:**
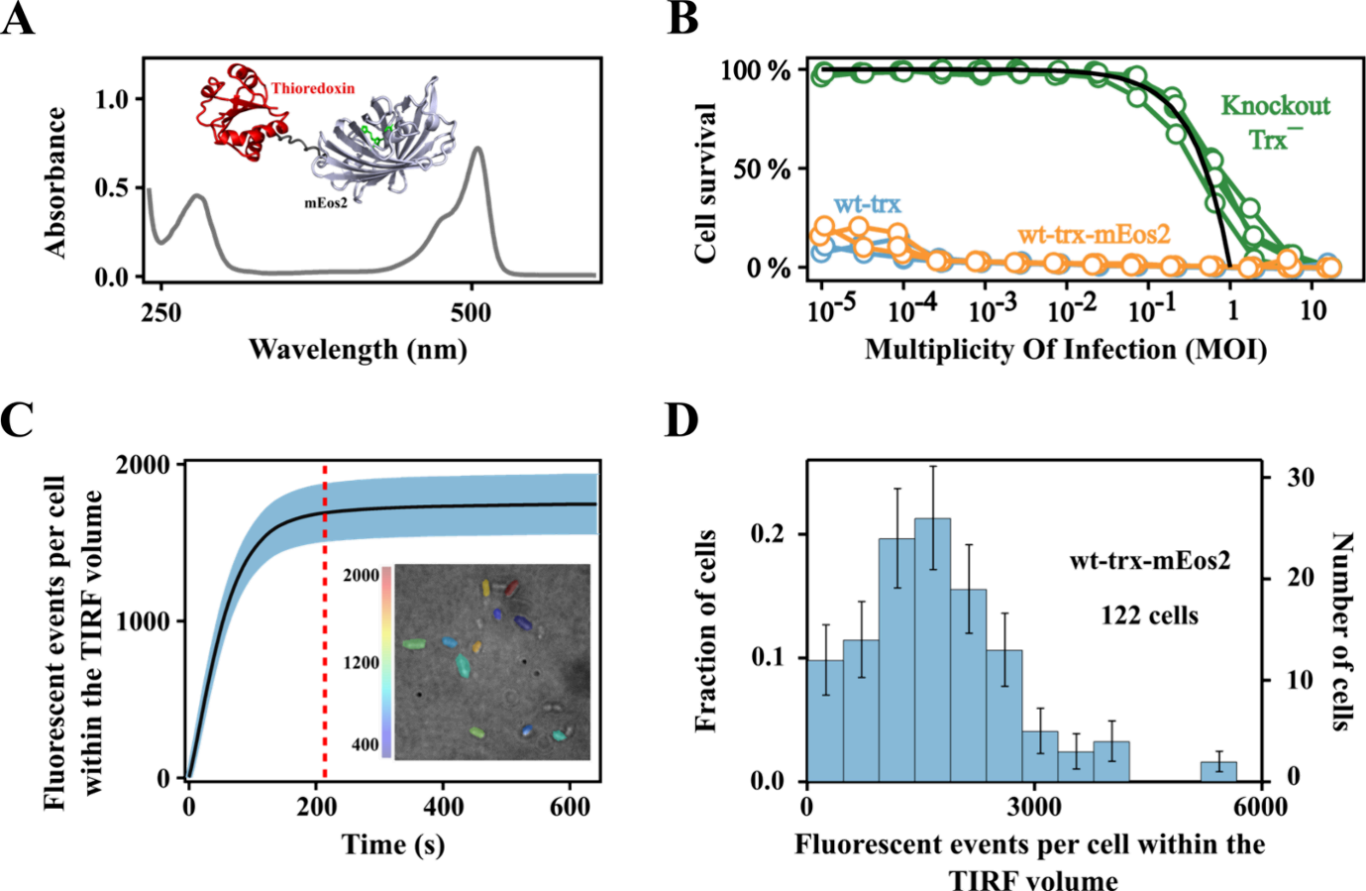
Single-molecule localization in cells transformed with wt-trx-mEos2. (A) Representative UV–vis spectrum and depiction of the 3D-structure of the wt-trx-mEos2 fusion construct. The construct was prepared following a cell growth protocol similar to that used for virus propagation and microscopy experiments. Three independent preparations led to a 506 to 280 nm absorbance ratio 1.45 ± 0.30, which is consistent with a 1:1 mEos2:thioredoxin stoichiometry (see Materials and Methods for details). (B) Propagation of phage T7 in cells transformed with wt-trx and wt-trx-mEos2. These experiments follow the same protocol as those of Figure 1C and show that the attached mEos2 does not preclude the death of all cells in virus experiments starting with low MOI values. (C) Cumulative number of fluorescent events per cell within the TIRF illumination volume derived from localization microscopy experiments with cells transformed with wt-trx-mEos2. The shadowed area is the 95% confidence interval, calculated by data bootstrapping. The dashed line indicates the time for quantification of fluorescent events at the plateau (*t* = 214s). The inset is a representative image with individual cells colored according to the number of fluorescent events detected. (D) Distribution of fluorescent events per cell within the TIRF volume at a representative time corresponding to the plateau (214 s). Data on 122 cells, involving 5 independent experiments from 4 biological replicas, were used to derive the distribution. For representation purposes, the experimental range of fluorescent events has been divided into 12 bins. The uncertainties bars at each bin are based on counting statistics. The structure of the fusion protein (panel A) is shown for illustration only and was constructed by connecting through the linker the structures of the partners taken from the Protein Data bank. No attempt was made to refine the structure of the linker, since it is expected to be highly flexible.

To quantify the number of thioredoxin molecules generated when knockout Trx^–^ cells are transformed with the wt-trx-mEos2 gene (strictly, to quantify the number of generated trx-mEos2 fusion constructs) we took advantage of the fact that photoconverted mEos2 moieties can be individually detected, provided that a suitably low intensity for the activating radiation is used. In a typical experiment, about 10 fixed cells in the same field of view are identified by transmission microscopy and the mEos2 moieties are simultaneously photoconverted using 405 nm constant illumination and imaged the photoconverted mEos2 at 561 nm using Total Internal Reflection (TIRF) configuration. The process is continued until all mEos2 molecules within the TIRF volumes are bleached (Supplementary Video 1). This experiment was repeated several times, with 3 different preparations, to yield a total number of 122 studied cells. The cumulative number of detection events increases with time until a plateau is reached when all mEos2 molecules have been photoconverted and detected. Figure 3C illustrates this behavior with the per cell average of detection events. Figure 3D shows that individual cells display different numbers of detection events at the plateau, likely reflecting the fact that gene expression is a stochastic phenomenon,^26^ although differences in the number of plasmid copies per cell may also contribute. In any case, most cells displayed substantial levels of thioredoxin expression, with an average value of 1737 ± 198 (95% confidence interval, CI) molecules per cell within the TIRF volume, as derived from the cumulative number of detections at the plateau.

It is important to note at this point that, in the experiments described above (Figures 3C and 3D), the TIRF depth was 150 nm, i.e., 6.5 times smaller than the relevant length of the *E. coli* cell (supplementary Figures S6, S7 and Materials and Methods). Independent FRAP (fluorescence recovery after photobleaching) experiments (supplementary Figure S6) showed that fluorescent proteins did not significantly diffuse within the cell cytoplasm in the time scale of the experiments. Consequently, only the molecules present within the TIRF illumination depth are detected and the number of trx-Eos2 copies measured in TIRF configuration needs to be scaled by a factor of 6.5 to estimate the total number of molecules present in the cell (supplementary Figure S7 and Materials and Methods). This results in about 11000 molecules per cell on average, a value on the order of the constitutive expression levels for thioredoxin in wild-type *E. coli*: ∼ 10000–20000 molecules per cell.^12,13^

### Single-Molecule-Microscopy Quantification of Host Factor Levels Generated through Codon Misreading

We now appended the mEos2 sequence to the ochre-containing thioredoxin gene (Figure 4A) and set out to quantify thioredoxin expression as we had previously done when no stop codon was present. That is, we transformed the knockout Trx^–^ cells with the ochre-trx-mEos2 gene, in such a way that any generated thioredoxin molecules necessarily result from codon misreading events. The attachment of mEos2 is not expected to change the number of generated thioredoxin molecules but only to provide a way to quantify them. This purpose would be served even if, due perhaps to steric interference, the presence of mEos2 precluded the interaction of thioredoxin with the viral DNA polymerase. Actually, we observed that cells transformed with ochre-trx-mEos2 could still sustain virus replication, although less efficiently than cells transformed with ochre-trx (Figure 4B and supplementary Figure S2).

**Figure 4:**
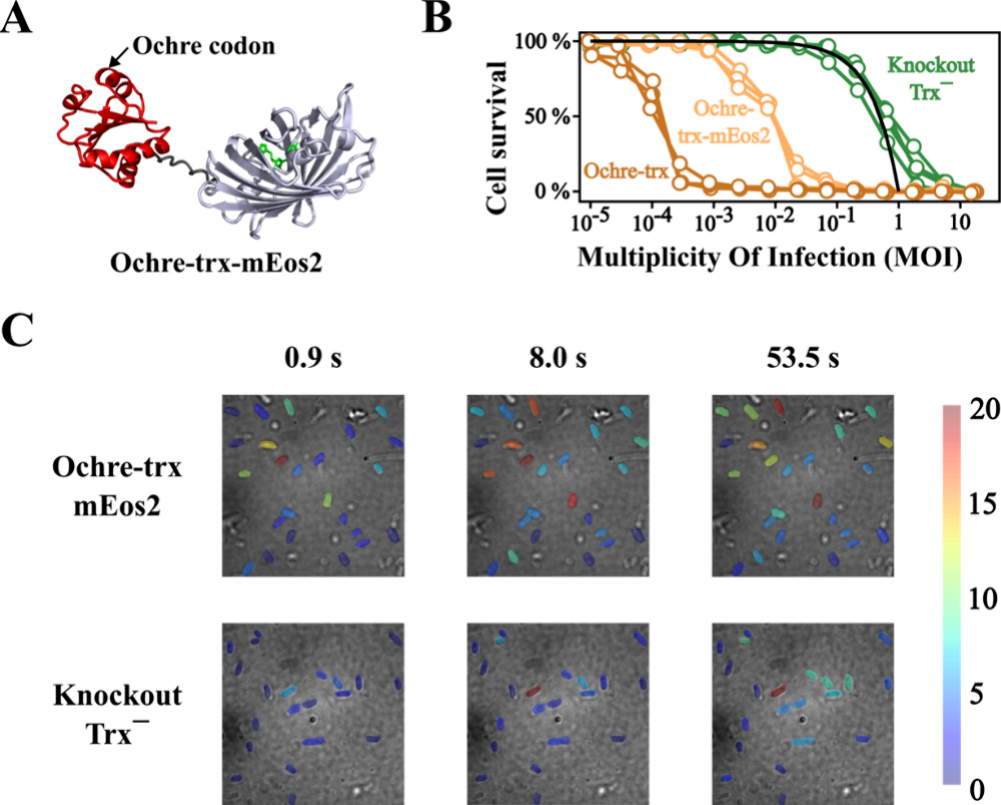
Single-molecule localization in cells transformed with ochre-trx-mEos2. (A) Depiction of the 3D-structure of the ochre-trx-mEos2 construct showing the position of insertion of the ochre codon. (B) Propagation of phage T7 in cells transformed with ochre-trx and with ochre-trx-mEos2. These experiments follow the same protocol as those of Figure 1C and show that, while the attached mEos2 impairs to some extent the cells capability to sustain virus propagation, it does not preclude complete cell death in experiments starting with low MOI values. (C) Representative images obtained in localization microscopy experiments involving cells transformed with ochre-trx-mEos2 and knockout Trx^–^ cells at increasing times during the photoactivation and acquisition sequence. Individual cells are colored according to the number of fluorescent events detected. These images are shown for illustration only. A quantitative analysis of the data is given in Figure 5. The structure of the fusion protein (panel A) is shown for illustration only and was constructed by connecting through the linker the structures of the partners taken from the Protein Data bank. No attempt was made to refine the structure of the linker, since it is expected to be highly flexible.

The profile of cumulative fluorescence events for the knockout cells (Figure 5A) can be interpreted as the sum of two quite different dependencies: an exponential-like increase that saturates at short times (i.e., an asymptotic exponential growth) plus a more gradual linear increase in the total number of events. None of these two dependencies can be attributed to the exogenous mEos2 protein, which is not expressed in the knockout cells. Actually, the exponential-like saturation is linked to the photobleaching of endogenous fluorophores upon illumination. For instance, porphyrins can be excited with 405 and 561 nm light and emit in the 600–700 nm range, partially overlapping with our detection range.^25^ Note that, as endogenous fluorophores are depleted, the signal saturates reflecting the exhaustion of fluorescent pool within the cells. A flat plateau is not observed, however, because of the linear increase contribution which is most likely due to scattering. Unlike fluorophore bleaching, scattering events occur independently of fluorophore availability and are primarily driven by the incident illumination power, resulting, at constant power, in a linear contribution to the cumulative count of events. Note that all the types of cells studied in this work are expected to give rise to endogenous fluorescence and scattering events. Yet, the contribution from these two processes would be much smaller than that arising from high levels of the exogenous mEos2. Therefore, endogenous fluorescence and scattering contributions are not apparent in profiles of cumulative fluorescence events for cells transformed with the wt-trx-mEos2 construct (Figure 3C). On the other hand, as elaborated in detail below, similar contributions from endogenous fluorescence, scattering and exogenous fluorescence are observed in cells transformed with the ochre-trx-mEos2 construct in which stop-codon misreading leads to exceedingly low levels of mEos2.

**Figure 5:**
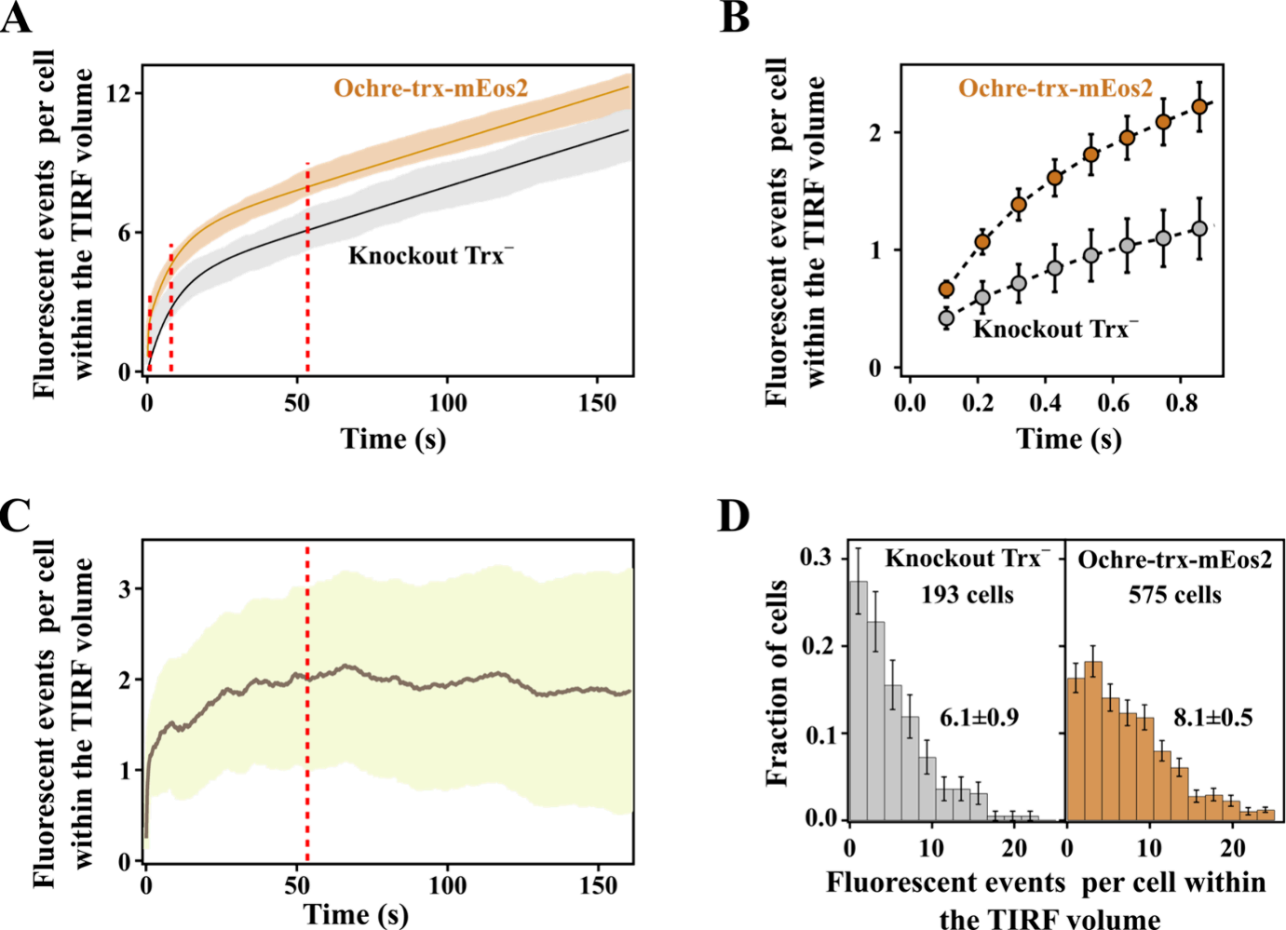
Quantification of thioredoxin molecules in cells transformed with ochre-trx-mEos2. (A) Number of fluorescent events per cell within the TIRF volume derived from photobleaching experiments with knockout Trx^–^ cells and with cells transformed with ochre-trx-mEos2. For knockout Trx^–^, data for 193 cells were obtained in 3 independent experiments involving 3 biological replicas. For ochre-mEos2, data for 575 cells were obtained in 5 independent experiments involving 5 biological replicas. The shadowed regions show the 95% confidence interval of the experimental data (calculated by bootstrapping). The solid lines are the cumulative exponential-linear fit (eqs 1 and 2, see text) fitted globally to the ochre-wt-mEos2 and knockout cells counts. (B) Blowup of the low-time region of the plot in A, highlighting the much faster initial increase of cumulative number of fluorescent events for the cells transformed with ochre-trx-mEos2 as compared with the knockouts. (C) Difference between the profiles for ochre-trx-mEos2 and knockout Trx^–^ shown in panel A. The solid line is the average value, and the shadowed area is the 95% confidence interval. The profile stabilizes at 2 ± 1 (95% CI) fluorescence events per cell within the TIRF volume. (D) Distribution of fluorescent events per cell within the TIRF volume for knockout Trx^–^ cells and for cells transformed with ochre-trx-mEos2. The distributions shown correspond to a time of 53.5 s. For representation purposes, the experimental range of fluorescent events has been divided into 12 bins. The uncertainties bars at each bin are based on counting statistics.

The profile of cumulative fluorescence events for the cells transformed with the ochre-trx-mEos2 construct are similar to those of the knockout cells (Figure 5A). That is, it shows an initial exponential-like increase that saturates at short times followed by a linear increase in the total number of events. There are, however, two clear differences between the two profiles. First, the number of fluorescent events per cell within the TIRF volume is consistently higher for cells transformed with ochre-trx-mEos2, as is apparent in Figure 5A. Second, the number of fluorescent events initially increases at a much faster rate for cells expressing ochre-trx-mEos2 as compared with the knockouts (Figure 5B). It is important to note that these differences are quite robust and reproducible. For cells expressing ochre-trx-mEos2, we thus performed experiments with 5 biological replicates, analyzing around 100 cells for each replicate (575 cells in total). Likewise, for knockout Trx^–^ cells, we performed experiments with 3 biological replicates, probing around 60 cells for each replicate (193 in total total). The agreement among the different experiments performed with each type of cells was excellent, as shown by the 95% CI in Figure 5A and 5B. There is no doubt, therefore, that total number of fluorescent events and the initial rates are higher for cells expressing ochre-trx-mEos2 as compared with knockout cells.

Overall, the contribution from mEos2 to the cumulative fluorescence profiles of cells transformed with ochre-trx-mEos2 while small, it is significant and clearly above experimental uncertainty. It follows that the exceedingly small number of trx-mEos2 constructs generated in cells transformed with ochre-trx-mEos2 may be quantified from the cumulative fluorescence profiles if the contribution of endogenous autofluorescence and scattering is eliminated by using a suitable data analysis protocol. To achieve this, we performed a global least-squares fitting of the two profiles of cumulative fluorescence events using the following eqs 1 and 2 that describe an asymptotic exponential growth with a linear slope:

$$N_{KO}=a_{0}\left(1-e^{-t/\tau_{0}}\right)+bt$$ 1

$$N_{OCHRE}=a_{1}\left(1-e^{-t/\tau_{1}}\right)+a_{0}\left(1-e^{-t/\tau_{0}}\right)+bt$$ 2

In these equations, *N*_KO_ and *N*_OCHRE_ are the cumulative numbers of fluorescent events for knockout Trx^–^ cells and cells transformed with ochre-trx-mEos2, respectively, and *t* is time. The exponential asymptotic terms, *a*_0_(1 – *e* ^–*t*/τ_0_^) and *a*_1_(1 – *e* ^–*t*/τ_1_^), describe exponential saturation due to the progressive bleaching upon illumination of endogenous and exogenous fluorophores, respectively, while the linear term *b*·*t*, captures the cumulative increase of registered events due to scattering. Note that the contribution from trx-mEos2 photoconversion/detection, i.e., the term *a*_1_(1 – *e* ^–*t*/τ_1_^), appears only in the equation for N_OCHRE_. In order to isolate this term, the two equations were simultaneously fitted to the profiles of N_KO_ and N_OCHRE_ imposing that the common parameters (*a*_0_, τ_0_ and *b*) must have the same values for the two profiles. The fit was visually excellent (Figure 5A) and led to the following values for the parameters describing the photoconversion term: a_1_ = 1.9 ± 0.7 (95% CI) fluorescent events per cell within the TIRF volume and τ_1_ = 0.2 ± 0.3 s (95% CI). The low value of τ_1_ is consistent with the time scale in which the initial increase in N_OCHRE_ takes place (Figure 5B). The value of *a*_1_ corresponds to the number of photoconvertible trx-mEos2 molecules per cell within the TIRF volume and translates into 12 ± 5 (95% CI) molecules per cell. Autofluorescence (due to endogenous fluorophores) and scattering, common to both ochre-trx-mEos2 and knockout cells, were characterized by *a*_0_= 3.9 ± 0.4 events per cell (95% CI) and *b* = 0.040 ± 0.002 (95% CI) events per cell and s. We note that autofluorescence showed a much slower increase, with a characteristic time τ_0_ = 8.4 ± 0.9 s (95% CI).

As a second approach, we simply subtracted the experimental curves and obtained a N_OCHRE_-N_KO_ difference profile (5C) that reached a plateau at 2 ± 1 (95% CI) fluorescent events per cell within the TIRF volume, in agreement with the a_1_ value calculated above. Finally, we calculated the distributions of cumulative fluorescent events for both types of cells at time at which the plateau in the N_OCHRE_-N_KO_ difference plot had been reached (53.5 s, Figure 5C). The distributions are clearly different (Figure 5D). In fact, the average numbers of fluorescent events per cell within the TIRF volume (Figure 5D) derived from the distributions are 8.1 ± 0.5 (95% CI) (cells transformed with ochre-trx-mEos2) and 6.1 ± 0.9 (95% CI) (knockout cells) and their difference is consistent with the a_1_ value derived from the global fittings based on eqs 1 and 2.

## Discussion

### On the Number of Thioredoxin Molecules That Enable Phage Replication

It is important to consider first the relation between the numbers of molecules detected by single-molecule localization microscopy (an average of 12 ± 5 molecules per cell) and the actual number of thioredoxin molecules that enable virus amplification. Several factors could potentially contribute to make the ∼ 12 average an overestimate of the actual number of enabling thioredoxin molecules per cell, namely, the possibility that the amino acid resulting from stop-codon misreading impairs proper thioredoxin folding, mEos2 blinking events^27^ and the possibility that translation is initiated at an alternative start codon^28–31^ after the engineered stop codon. The contributions of these factors, however, are expected to be comparatively minor (see the relevant section in Supplementary Discussions for details). In any case, overestimation would strengthen the main conclusions of this study even further, simply because it would imply that the number of thioredoxin molecules enabling virus amplification is even smaller than ∼ 12.

On the other hand, localization microscopy could underestimate the number of thioredoxin molecules present in the cell if some of the mEos2 proteins fail to fold properly to the photoconvertible state, thus decreasing the photoconversion efficiency. However, mEos2 has been reported to have the largest photoconversion efficiency among commonly used photoconvertible and photoactivable proteins (over 60%).^32^ In our hands, mEos2 displays a high folding efficiency and, indeed, UV–vis spectra of our purified wt-trx-mEos2 fusions (Figure 3A) indicate an approximately 1:1 fluorescent-mEos2: thioredoxin stoichiometry.

Finally, it is important to note that, since successful phage T7 amplification in a cell leads to an average of hundred or more of new virions,^22^ propagation could be triggered by a fraction of the cells that express numbers of thioredoxin molecules above the average value, i.e., by cells at the high tail of the number of molecules distribution (see Figure 5D). These uncertainties notwithstanding, the single-molecule localization experiments confirm that phage T7 replication does occur in a population with about 10 host factor molecules per cell, as we originally inferred from the fact that the presence of the ochre codon does not abolish replication.

### A Rationale for Phage Replication at Exceedingly Low Host-Factor Levels

Phage replication based on a number of thioredoxin molecules per host cell on the order of ten or a few tens is astonishing. Yet, we show here that a reasonable and convincing explanation for this result can be put forward on the basis of the following two notions: 1) that, as a consequence of the high affinity of thioredoxin for the viral DNA polymerase, thioredoxin-polymerase complexes are formed, even if the number of thioredoxin molecules per cell is very low; 2) that a few polymerase-thioredoxin complexes per cell can reasonably sustain DNA replication at the expected elongation rates. These two notions are expounded in some detail below:

The interaction of viral DNA polymerase with thioredoxin is very tight and dissociation constant of 5 nM has been reported for the complex.^14,15^ Since the volume of an *E. coli* cell is about 1.3·10^–15^ liters (see Materials and Methods), a simple calculation using the Avogadro’s number yields that 5 nM is equivalent to just about 4 thioredoxin molecules per cell. Therefore, copy numbers on the order of ten or a few tens thioredoxin molecules imply cellular thioredoxin concentrations clearly above the dissociation constant for the interaction between thioredoxin and the viral DNA polymerase. Consequently, about a few tens of DNA polymerase-thioredoxin complexes would be formed in each host cell (see supplementary Figure S8). Furthermore, even a few functional thioredoxin-polymerase complexes should suffice to sustain virus replication. Phage T7 has a genome size of about 4·10^4^ bp^22^ and, at an elongation rate of several hundred nucleotides per second,^33^ a few tens of functional DNA polymerase-thioredoxin complexes could produce about a hundred copies of the phage genome in just a few minutes. Note that the burst size of phage T7 is actually about 100 virions.^22^

Admittedly, the illustrative calculations provided in the preceding paragraph are based upon a value of the dissociation equilibrium constant that was determined in *in vitro* assays with purified proteins.^14^ See the relevant section in Supplementary Discussions for a justification of the use of an *in vitro* dissociation constant value to evaluate intracellular polymerase-thioredoxin interactions. In particular, the role of thioredoxin interactions with other cellular proteins^16^ and that of macromolecular crowding in the intracellular milieu^34^ are briefly discussed.

Finally, we note that, for the molecular rationale proposed in this section to be fully convincing, we need to provide an evolutionary narrative for the fact that the interaction between thioredoxin and the viral DNA polymerase seems to be much tighter than seemingly required by the normal cellular thioredoxin levels. A convincing evolutionary interpretation of the tight polymerase-thioredoxin interaction can be easily constructed based on the fact that thioredoxin functions as a processivity factor for the polymerase. Binding of thioredoxin suppresses this hopping on and off of the polymerase from the DNA and converts the enzyme in a highly processive polymerase able to synthesize long DNA stretches.^9,33^ This function of thioredoxin as a processivity factor requires that the polymerase-thioredoxin complex displays a sufficiently long residence time. This is the average time a complex exists before dissociating, it is given by the inverse of the dissociation rate constant,^35^ 1/k_OFF_, and reflects, therefore, the kinetic stability of the complex. The dissociation constant for the thioredoxin-polymerase complex is related to the rate constants for association and dissociation through K_D_=k_OFF_/k_ON_. Therefore, a very low value for the dissociation equilibrium constant, K_D_, likely results from natural selection for a very low value of the dissociation rate constant, k_OFF_, as required to guarantee a long residence time, 1/k_OFF_. A more detailed account of this interpretation is provided in the corresponding section of the Supplementary Discussions section.

### On the Role of Gene Expression Errors in Virus Adaptation

Crucial biomolecular interactions between viral and host biomolecules may need occur only a few times per host cell to enable virus propagation. This notion could perhaps be reasonably inferred from the current understanding of the molecular mechanisms of virus replication, but it is experimentally demonstrated in this work. The capability of viruses to use biomolecules at very low copy numbers supports that viruses can take advantage of pre-existing low-level protein diversity generated by gene expression errors. Certainly, nonprogrammed gene expression errors are to a large extent random, although not necessarily fully random.^36,37^ Yet the molecular diversity they generate may be simply overwhelming. For instance, using the recent estimate^21^ of about 20% for the fraction of protein molecules bearing phenotypic mutations as a result of gene expression errors, it is easily calculated that, out of 10^5^ copies per cell of a given representative protein,^38^ around 20000 would be variants harboring phenotypic mutations. This number of variants is about 3 times the total number of possible single amino acid replacements (19 × 375) in a human protein of median length.^39^ That is, random gene expression errors may lead to a good coverage of, at least, all the single mutant variants of a protein. Overall, phenotypic mutations occur at high frequency and are highly diverse. It is not surprising, therefore, that phenotypic mutations have been proposed to play various roles in adaptive evolutionary trajectories despite being noninheritable.^40–42^ In the particular case of viruses, the fact that the infection of a single host cell leads to a large number of virions suggests that, both viral and host protein variants carrying phenotypic mutations may enable virus propagation in some scenarios.^42^ The statistical plausibility of this scenario is supported by order of magnitude estimates on the variants of proteins essential for the replication SARS-CoV2 and influenza viruses that we provide in the Supplementary Discussions. How specifically phenotypic mutations may contribute to viral adaptation is elaborated in the next section using the phenomenon of cross-species transmission as an illustrative example.

### Cross-Species Transmission as a Potential Example of the Role of Expression Errors

Viruses occasionally jump between species, sometimes with disastrous consequences for the new hosts. Cross-species transmission, however, is puzzling from both, the molecular and the evolutionary points of view. Viruses interact extensively with the molecular machinery of the host.^8^ This includes recruiting host proteins for processes critical for virus propagation, blocking and evading antiviral factors and generally repurposing the host molecular machinery for virus proliferation. For instance, an experimental study into the influenza virus-host interactome^43^ identified interactions of various viral proteins with several hundred host factors. A virus involved in cross-species transmission (a “jumping virus” so to speak) must be able to replicate in two different hosts simultaneously which implies that it must be able to establish a very large number of biomolecular interactions simultaneously in both, the old host and the new host. The jumping virus is certainly adapted to the molecular environment of the old host. However, it needs to establish all the crucial interactions also in the new host, that is, in a molecular environment to which it is not yet adapted, and which may substantially differ from that of the old host. Note in particular that homologue proteins in different organisms, while likely sharing the same overall fold, will differ in sequence and, therefore, in the chemical features of the exposed surfaces available for interaction. Consequently, the fact that the virus can establish a given crucial interaction in the old host does not imply that it will be able to immediately establish the same interaction in the new host. The difficulty is magnified if, as it seems likely, many such interactions involving different viral proteins and many different host factors need to be established.

The preceding paragraph describes a kind of problem that is well-known in evolutionary theory. It can be posed in the following way. It is easy to understand how natural selection enhances a functionality that confers a survival advantage to the organism, but it is difficult to understand how a completely new functionality (i.e., an innovation) arises, given that natural selection has no foresight^4^ and, therefore, cannot act on a function before this function exists. Specifically, for a virus to be able to cross species, it must already display some degree of molecular adaptation to the new host, but natural selection will lead to adaptation to the new host only after the virus has jumped and it is replicating in the new host. However, phenotypic mutations may provide a way to bypass this Catch-22 situation. As illustrated in this work, crucial virus-host biomolecular interactions may be enabled by just a few protein molecules. Therefore, the wide diversity of protein variants generated by gene expression errors could provide an extensive capability to establish/avoid biomolecular interactions in different environments. Certainly, replication based on a small number of enabling protein molecules that occur statistically among the diversity of variants generated through gene expression errors, will likely be inefficient and transient. Yet, it will allow the virus to initially survive in a new environment (a new host, for instance) and will thus give natural selection a chance to act and generate adaptation at the genetic level. It is to be noted that, in this context, the fact that gene expression errors are not inheritable may actually be an advantage, as it implies that the generated protein diversity will not be “pruned” by natural selection for adaptation to the current environment of the virus and, therefore, will remain available for the virus to meet future challenges.

Certainly, the narrative provided in the preceding paragraph embodies a hypothesis which, albeit reasonable, will need to be sustained (or rejected) by future experimental work. The general point is, however, that this work supports the possibility that gene expression errors may play roles in virus adaptation processes beyond the known instances of programmed recoding. Some suggestions can be offered regarding how the role of gene expression errors on virus adaptation could be experimentally addressed. One interesting possibility in this context is that phenotypic mutations caused by transcription errors are particularly relevant. It must be noted first that difference between genetic mutations and phenotypic mutations linked to transcription errors applies even to RNA viruses, since the viral RNA-polymerase would generate both genomic RNA to be encapsulated in the virions and mRNAs to be used in protein synthesis. Certainly, the transcription error rate is low as compared with the translation error rate.^18^ However, transcription errors may have a stronger impact at the protein level because each mRNA molecule is typically translated many times.^44^ Next-generation sequencing methodologies with the capability to determine mutations present in RNA at very low level are available.^45^ Furthermore, transcription errors are known to be far from completely random.^36,37^ It would seem then feasible to determine the landscape of transcription errors for viral (and host) proteins and to assess the extent to which the most prevalent phenotypic mutations enable biomolecular interactions crucial for virus propagation. Work along these lines focusing on the hemagglutinin of influenza virus is currently under way in our lab.

## Material and Methods

### Cells

*E. coli* expresses thioredoxin 1 (which we refer throughout this work simply as “thioredoxin”) and thioredoxin 2, which is induced under oxidate stress and includes a zinc-binding domain.^10^ To our knowledge, thioredoxin 2 does not act as a processivity factor for the viral DNA polymerase. In any case, the knockout Trx^–^ strain used in this work (originally a gift from Jon Beckwith, Harvard Medical School) is deficient in both thioredoxin genes, *trxA* and *trxC*. Specifically, this work uses the strain FA41, which is deficient in the two thioredoxins described in *E. coli*: thioredoxin 1 (thioredoxin) and thioredoxin 2. We refer to this strain as *E. coli* Trx^–^. This strain can grow, albeit more slowly than the corresponding wild-type DHB4 strain, likely because the glutaredoxin pathway substitutes to some extent the functions of thioredoxin. Genes encoding thioredoxin and thioredoxin-fluorophore constructs (both with and without stop codons at position 11 in the amino acid sequence) were synthesized with a His-tag at the C-terminal and cloned into pET30a­(+) plasmid with kanamycin resistance (GenScript Biotech). Transformation of knockout Trx^–^ cells and other details are essentially as we have previously described.^10^

Transformed cells were stored as glycerol stocks and only retrieved and grown immediately before experiments. Furthermore, we checked regularly through sequencing that cells harboring the constructs bearing a stop-codon were stable in the sense that a genetic mutation that replaced the stop codon had not occurred (see Supplementary Figure S9). More generally, the possibility that our results are linked to compensatory genetic mutations that eliminate the stop-codon, rather than to gene expression errors, can readily be ruled out on the basis of several straightforward arguments. First, as it is well-known, genetic mutations are several orders of magnitude less probable than gene expression errors.^19^ Second, if a genetic mutation that rescued the stop codon had occurred prior to a virus propagation experiment and dominated the population, then the propagation profiles for the stop codon constructs would have resembled those for the wild-type protein and this is not observed in Figure 1C. It is important to note here that propagation profiles differing substantially from that corresponding to the WT construct were obtained for three replica experiments with three different constructs, i.e. Nine experiments in total (Figure 1C). Third, the trend observed for the propagation profiles (opal > amber > ochre) corresponds to the known readthrough propensities.^5^ Fourth, if the compensatory genetic mutation occurred during the virus propagation experiments, it would lead to cells that would be easily killed by the virus and the mutation would not propagate; that is, there is no selection for compensatory genetic mutations during the virus propagation experiments (if anything, there would be selection against those mutations).

Purification of the Trx-mEos2 construct for determination of its UV–vis spectrum was carried by Ni-NTA affinity chromatography, followed by passage through PD10 columns to obtain protein solutions in Hepes 50 mM, pH 7. Protease inhibitors were added in all steps of the purification. Three independent purifications were carried out, leading to an average value of 1.45 ± 0.30 for the 506 nm vs 280 nm absorbance ratio. The absorbance peak at 506 nm is due to the fluorophore and has a reported extinction coefficient of 5.6·10^4^ M^–1^cm^–1^.^6^ The absorbance peak at 280 nm reflects protein contributions from both mEos2 and thioredoxin. Published spectra indicate extinction coefficients at 280 nm of about 2.5·10^4^ M^–1^cm^–1^ for mEos2 ^6^ and of 1.4·10^4^ M^–1^cm^–1^ for thioredoxin.^46^ The fusion trx-mEos2 construct, therefore, is expected to have an extinction coefficient of 3.9·10^4^ M^–1^cm^–1^ at 280 nm and show a 506 nm vs 280 nm ratio of 1.44 in agreement with our experimental value of 1.45 ± 0.30.

### Plaque Assays

Plaque assays were carried out as previously described.^47^

### Lysis Experiments

Lysis experiments were performed using a simplified adaptation of a previously employed protocol.^47^ Briefly, overnight preinocula were incubated in LB medium at 37 °C, which, for transformed cells, had been supplemented with kanamycin (50 mg mL^–1^). Cultures were diluted 1/200 in fresh medium and, when the absorbance at 600 nm had reached 0.25–0.30, microliter volumes of virus stock solution were added to the desired Multiplicity of Infection (MOI). Cultures were incubated for 1 h at 37 °C and absorbance was measured again. In experiments with very high MOI values, the absorbance values at 600 nm measured after 1 h were on the order of 10^–2^-10^–3^, indicating that essentially all cells had been killed. The survival cell fraction at each given MOI was calculated from the absorbances at 600 nm using the following eq 3:

$$\%\;cell\;survival=\frac{\left(A-A_{L}\right)}{\left(A_{0}-A_{L}\right)}\times 100$$ 3

where A_0_ and A are, respectively, the initial absorbance and the absorbance at 1 h in one experiment corresponding to a given MOI value, and A_L_ is the (very low) absorbance at 1 h in another experiment in which a very high MOI value was used and essentially complete cell death was attained after 1 h. MOI values were calculated from the concentration in the virus stock solution in plaque-forming-units (PFU) per unit volume. This concentration was calculated for each amplified phage using plaque assays with a DHB3DE3 *E. coli s*train as previously described.^10^

### Single Molecule Experiments

Bacterial cultures were grown for microscopy experiments on a sterilized chambered 1.5H glass bottom coverslip with 8 individual wells coated with poly-l-lysine. 350 μL of a 1/1000 dilution from an over day preinoculum, that had reached exponential growth phase, were transferred to each well. These cultures were incubated overnight and protected from light at RT. The next day, the wells were washed to eliminate floating and nonimmobilized bacteria before fixation with 4% paraformaldehyde and quenched with sodium borohydride (NaBH_4_) to decrease autofluorescence. Finally, 250 μL of a 1/1000 dilution in PBS of 80 nm gold colloid beads were added to the wells to allow image drift correction when necessary.

Experiments were carried out on a TIRF microscope (Leica Microsystem), with an oil-immersion objective HC PL APO 160x 1.43 NA, an Orca Flash 4.0 sCMOS camera (Hamamatsu) and high-power lasers suitable for single molecule localization microscopy techniques. An additional high pass filter (OG530) was located at the microscope condenser to prevent spontaneous mEos2 photoconversion during sample manipulation and focusing.

Single images of mEos2 before photoconversion were acquired at 488 nm at minimal power to prevent mEos2 copies from bleaching. mEos2 was then photoconverted by continuously illuminating the sample at 405 nm throughout the length of the experiment using either 1 or 0.1 mW/mm^2^ power density depending on the sample. Samples were simultaneously imaged at 561 nm (20 W/mm^2^) using a multiband bandpass filter (excitation: 397–413 nm; 481–495 nm; 550–566 nm; 627–643 nm. Emission: 420–480 nm; 500–550 nm- 580–630 nm; 660–860 nm) at the microscope and additional bandpass filters (Semrock FF01–512/25–25; FF01–630/92–25) to further filter out 405-induced autofluorescence and scattering. The exposure time was 100 ms. All images were acquired on TIRF configuration (150 nm penetration depth) with 102 nm pixel at the sample plane (256 × 256 pixels). The total length of the imaging sequence was either 5 or 10 min (depending on the sample).

### Confocal Microscopy

Bacterial cultures were grown in conditions similar to those used in experiments of single molecule and experiments with living cells, without sample fixation nor quenching, and were imaged by confocal microscopy. Images were acquired using a Leica STELLARIS 5 confocal microscope (Leica Microsystems) with an oil immersion HC PL APO 63x 1.40 NA objective (Leica Microsystem). Samples were excited at 488 nm using either 4 W/mm^2^ or 20 W/mm^2^ at the sample plane depending on the sample. Fluorescence emission was collected using a hybrid (HyD) S detector in the 498 nm-620 nm spectral window, 52 nm pixel size, and using the same gain for all samples (2.5%).

### Flow Cytometry Analysis

For flow cytometry experiments, bacterial cultures were grown from glycerol stocks, and they were cultured overnight in LB medium supplemented with kanamycin (50 mg mL^–1^), if necessary, at 37 °C. The next day, optical density was measured and samples were diluted to 0.3 OD600 in filtered PBS buffer, and 300 μL of diluted sample were transferred to flow cytometry tubes for the analysis. trx-eGFP expression was analyzed using the Digital Analyzer GALLIOS cytometer (Beckman Coulter). Results were analyzed with the Kaluza software (Beckman Coulter).

### FRAP Experiments

Bacterial cultures were grown and prepared for analysis as previously described for single molecule experiments. Fluorescence recovery after photo bleaching (FRAP) experiments were performed on a Leica STELLARIS 8 STED 3X confocal microscope (Leica Microsystems) using a water immersion HC PL APO CS2 63x 1.20 NA objective. FRAP was performed using the FRAP AB mode of the Leica LAS X software. Prebleaching and postbleaching imaging were performed at 491 nm and fluorescence emission was collected using a hybrid spectral (HyD S1) detector in the range of 499 nm-594 nm. Bleaching was achieved at 491 nm with a 30 W/mm^2^ laser power density, using zoom in mode. For every cell a 0.07 μm^2^ bleaching area was defined, and the FRAP protocol was established as follows: prebleaching one-image acquisition, followed by 15 s bleaching and 80-s postbleaching recovery.

### Single-Molecule Data Analysis

Molecules were localized using the Thunderstorm^48^ plugin for ImageJ. Camera parameters were specifically configured with a pixel size of 101.6 nm, 0.46 photoelectrons per A/D count, and a base level of 1650 ADU. Fitting parameters used were difference-of-Gaussians filter, local maximum detection of single molecules and integrated Gaussian weighted least-squares fitting. Postprocessing steps include drift correction by fiducial markers and frame-merging (unlimited).

Cells were automatically identified and segmented based on the phase-contrast and 488-excitation (before photoconversion) images using a deep-learning 2D-segmentation algorithm (Misic).^49^ The resulting segmentation was subsequently examined, the cell contours were amended where needed and interfaced them with Fiji^50^ using the Roifile Python Library.^51^ We then assigned every localization in the PALM image sequence to its corresponding cell and built the empirical cumulative sum of fluorescent events per cell. Finally, we fitted a cumulative (double/single) exponential – linear model to the identifications cumulative sum, weighting the optimization with the uncertainty of each point. To estimate the uncertainty of the fitted parameters (a_0_, a_1_, τ_0_, τ_1_, b), we randomly resampled seven times (without replacement) the ochre and knockout data sets (575 and 193 cells, respectively). This way, we produced seven subsets of approximately 82 (ochre) and 27 (knockout) cells each. We then global-fitted the equations to the subsets as explained above. We finally computed the uncertainty of the fitted parameters as the standard error of the mean of the obtained seven values and multiplied this value times 1.96 to derive the 95% confidence interval. This resampling procedure was repeated at least five times with identical results within the experimental uncertainty. Image analysis and fitting algorithms developed for this study are available at https://github.com/cnbbiophot.

### Determination of the Cell Volume

We measured the long (*L* = 2.02 ± 0.04 μm) and short (*w* = 0.96 ± 0.01 μm) cell dimensions from the phase contrast images using the Fit Ellipse tool in Fiji. We measured 510 cells and assumed sphero-cylinder morphology (eq 4) to compute the cell volume, resulting in *V =* 1.30 ± 0.06 fl. All errors are 95% CI.

$$V=\frac{\pi w^{2}}{4}\cdot \left(L-\frac{w}{3}\right)$$ 4

## Supplementary Material









## Data Availability

Raw experimental data are available from the authors upon reasonable request.

## Notes

Additional experimental details and analyses; additional discussions on the interpretation and implications of the results reported (PDF)
Representative video illustrating the experiment of single-molecule localization microscopy: knockout (MP4)
Representative video illustrating the experiment of single-molecule localization microscopy: ochre (MP4)
Representative video illustrating the experiment of single-molecule localization microscopy: wild type (MP4)
