# Supplementary material for: Virus Propagation Linked to Exceedingly Rare Gene-Expression Errors: A Single-Molecule Microscopy Demonstration
Source: ACS Chem Biol. 2025 Oct 10;20(11):2720–32. doi: 10.1021/acschembio.5c00638 (PMC12645430; doi:10.1021/acschembio.5c00638)
Supplement: Supplementary file 1 [file cb5c00638_si_001.pdf]

Supporting Information for:

Virus propagation linked to exceedingly rare gene-expression errors: a single-molecule microscopy demonstration

Raquel Luzón-Hidalgo[1], Gianluca d'Agostino[2], Valeria A. Risso[1], Asuncion Delgado[1], Beatriz Ibarra-Molero[1], Luis A. Campos[3], Jose Requejo-Isidro[2,4] & Jose M. Sanchez-Ruiz[1]**

[1] Departamento de Quimica Fisica. Facultad de Ciencias, Unidad de Excelencia de Quimica Aplicada a Biomedicina y Medioambiente (UEQ), Universidad de Granada 18071 Granada, Spain.

[2] Centro Nacional de Biotecnología (CNB), CSIC, 28049 Madrid, Spain.

[3] IMDEA-Nanociencia, Ciudad Universitaria de Cantoblanco, 28049 Madrid, Spain.

[4] Unidad de Nanobiotecnología, CNB-CSIC-IMDEA Nanociencia Associated Unit, 28049 Madrid, Spain.

*Corresponding authors: jose.requejo@csic.es; sanchezr@ugr.es

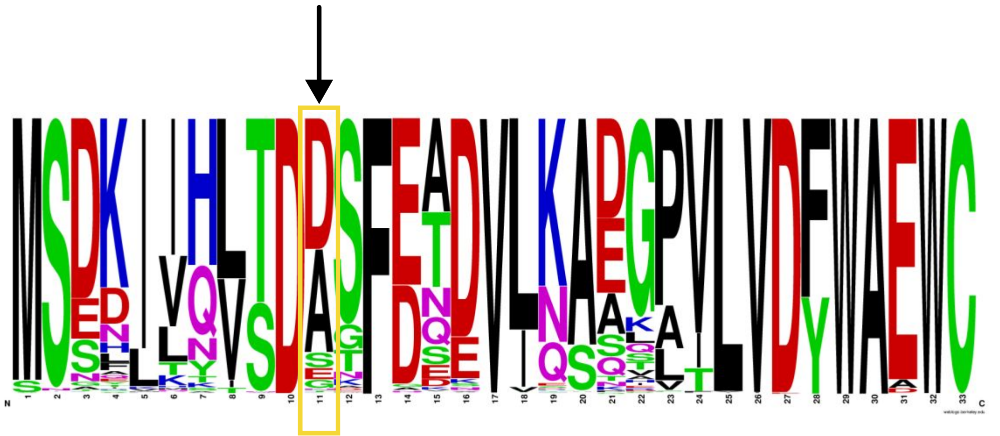

Supplementary Figure S1. Sequence logo for thioredoxin. A blast search was performed using the sequence of *E. coli* thioredoxin. The sequences with identity with the query higher than 70 % were retained for residue frequency calculation. Position 11 is exposed to the solvent in the 3D-structure of thioredoxin (Figure 1B). An aspartate is present at position 11 in *E. coli* thioredoxin. However, the frequency analysis summarized in the sequence logo indicates that position 11 in thioredoxins can admit other amino acid residues. Position 11 was selected for stop-codon insertion.

A

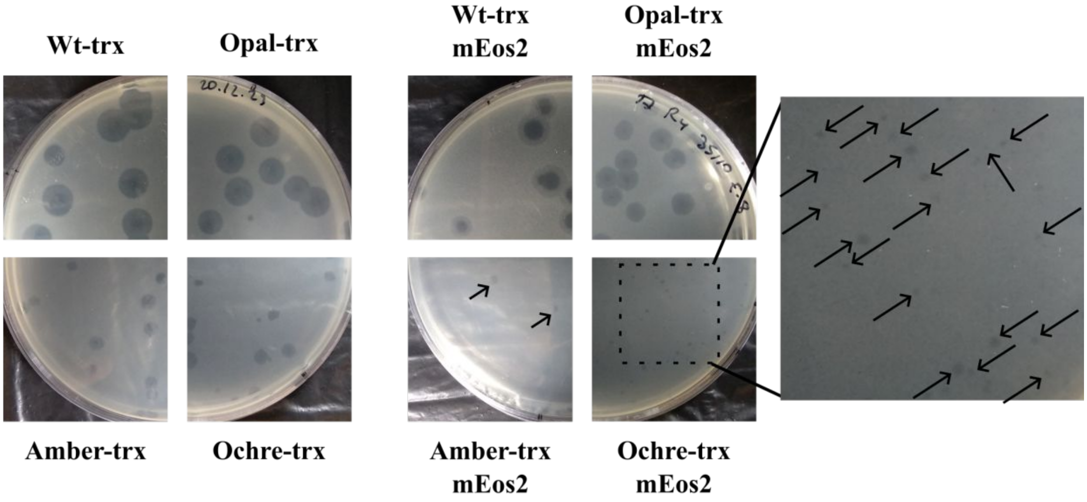

B

SOLUTION / STRAIN	PFU/mL A	PFU/mL B	PFU/mL C
Knockout trx$^-$	0	0	0
wt-trx	$(2.6 \pm 0.6) \times 10^9$	$(7.3 \pm 0.9) \times 10^9$	$(8.2 \pm 1.1) \times 10^9$
wt-trx-mEos2	$(2.0 \pm 0.7) \times 10^9$	$(5.2 \pm 2.6) \times 10^9$	$(6.1 \pm 0.1) \times 10^9$
Opal-trx	$(1.4 \pm 0.5) \times 10^9$	$(6.5 \pm 0.7) \times 10^9$	$(8.4 \pm 0.5) \times 10^9$
Opal-trx-mEos2	$(5.2 \pm 3.0) \times 10^8$	$(2.3 \pm 0.4) \times 10^9$	$(3.6 \pm 0.6) \times 10^9$
Amber-trx	$(1.2 \pm 0.2) \times 10^9$	$(3.9 \pm 0.7) \times 10^9$	$(3.6 \pm 0.8) \times 10^9$
Amber-trx-mEos2	$(9.0 \pm 7.9) \times 10^7$	$(4.6 \pm 0.6) \times 10^8$	2.00×10^8
Ochre-trx	$(1.2 \pm 0.3) \times 10^9$	$(1.3 \pm 0.4) \times 10^9$	$(2.1 \pm 1.2) \times 10^9$
Ochre-trx-mEos2	$(5.9 \pm 3.0) \times 10^6$	$(1.2 \pm 0.2) \times 10^7$	$(2.6 \pm 0.6) \times 10^7$

Supplementary Figure S2. Determination of plaque-forming units (PFU) for phage solutions. (A) Representative examples of T7 plaque formation after inoculating various hosts with the phage. The labels indicate the gene with which the knockout Trx$^-$ had been transformed. Plaques observed with cell transformed with ochre-trx-mEos2 are small and blowup is provided. (B) Number of PFUs per millilitre for 3 phage T7 solutions (labelled A, B and C) derived from 3 independent amplifications. Various strains were inoculated with the virus solutions. Except for the knockout Trx$^-$, the strains are identified by the gene used in the transformation. Values of PFU/mL for the phage solutions were calculated from the numbers of plaques upon serial dilutions, as described in Luzon-Hidalgo et al. (2021). Average values and standard deviations from three determinations are given.

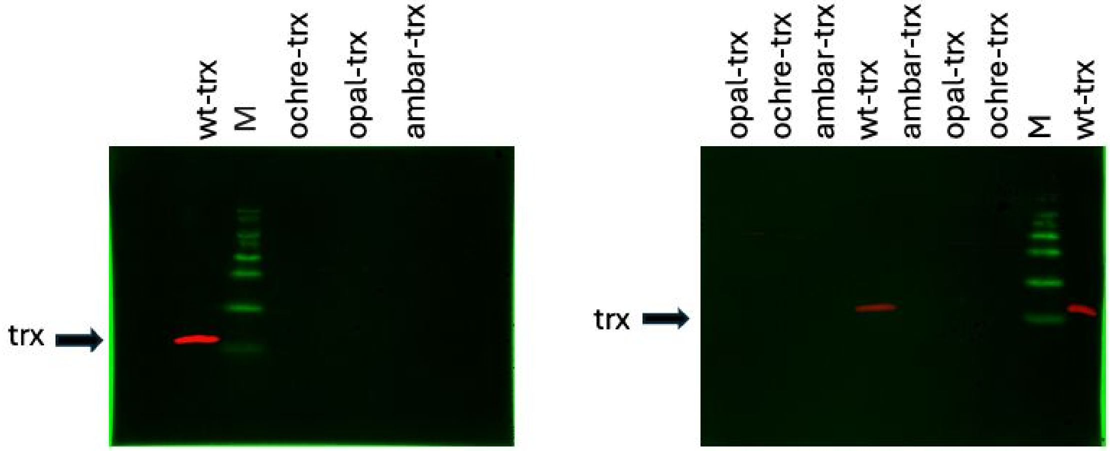

Supplementary Figure S3. Attempts to use Western blots to determine thioredoxin expression levels in Trx⁻ cells transformed with wt-trx, opal-trx, amber-trx and ochre-trx genes. Bacterial cultures were grown from glycerol stocks and were subsequently cultured overnight in LB medium supplemented with kanamycin (50 ug/mL) at 37 °C, harvested by centrifugation, resuspended in a lysis buffer (PBS and Protease inhibitor cocktail) and sonicated in short bursts to prevent overheating, and disrupted by sonication. Total protein samples were prepared by combining equal volumes of the disrupted cell suspensions and 2 × sample buffer. Typically, 10 μL of cell suspensions with about 10^{10} cells per millilitre were loaded. Western blots were revealed with *s* Thioredoxin 1 Polyclonal Antibody produced in rabbit (Thermo Fischer scientific PA5-117517) and Horseradish Peroxidase (HRP)-conjugated goat anti-rabbit antibodies (Santa Cruz Biotechnology). After chemiluminescence (ECL Bio-Rad) detection, the membrane was photographed. The results of three independent experiments are shown. Note that, while thioredoxin produced in cells transformed with the wt-trx construct is easily detected, no thioredoxin generated through stop-codon misreading is detected. This result could have been anticipated. Note that cells transformed with wt-trx are expected to express thioredoxin at copy numbers of thousands or tens of thousands per cell and, as shown by a simple calculation, 10 μL of a cell suspension with about 10^{10} cells per millilitre contains on the order of tens of nanograms of thioredoxin, within the typical range for Western Blots. On the other hand, cells transformed with the constructs that include stop codons express thioredoxin at much lower levels. In these cases, ensuring that tens of nanograms of thioredoxin are loaded would require using unrealistically large amounts of cell suspension.

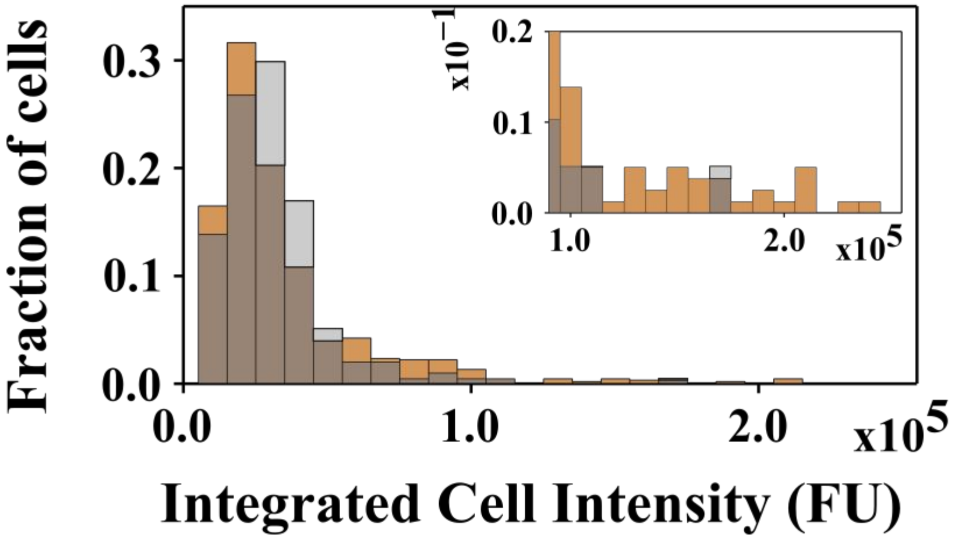

Supplementary Figure S4. Distribution of fluorescence intensity for cells transformed with ochre-trx-eGFP. The data (ochre) are shown superimposed with those corresponding to the knockout Trx^{-} cells (grey). The distributions show that the fluorescence levels upon transformation with ochre-trx-eGFP are similar, for almost all cells, to those observed with the knockout *E. coli* Trx^{-} cells, indicating that the contributions from endogenous autofluorescence and eGFP are comparable. Yet, the blowup of the high fluorescence intensity region (inset) shows a prevalence of ochre-trx-eGFP transformed cells and supports that a small fraction of these cells expressed eGFP to a detectable level in these experiments. Overall, however, it does not appear possible to use eGFP fluorescence to arrive at an estimate of the average number of molecules generated per cell.

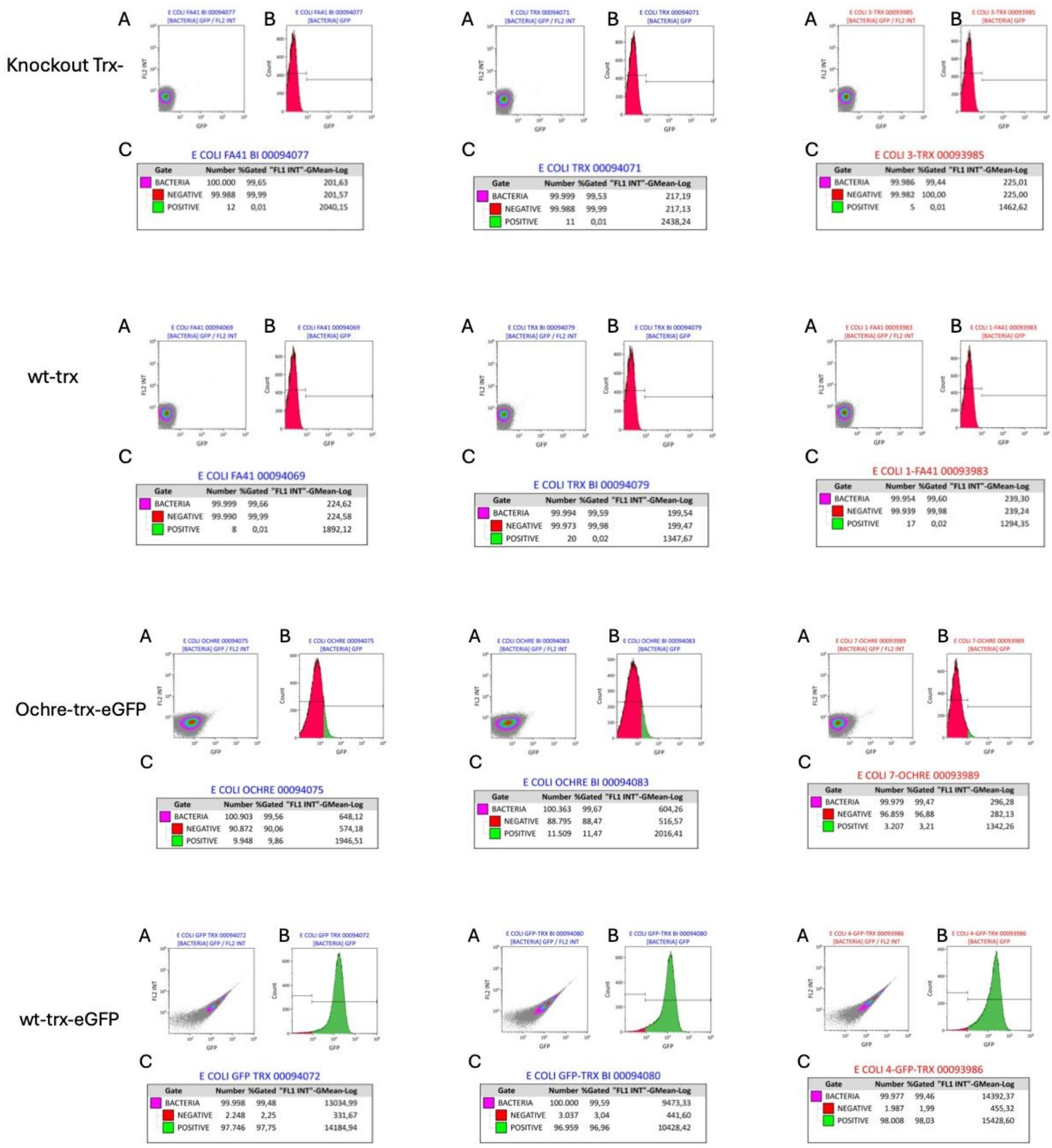

Supplementary Figure S5. Results from flow cytometry experiments in the emission window of eGFP. (A) Dot plots describing the cell population distributions derived from flow cytometry experiments. (B) Histogram plots of eGFP fluorescence intensity distribution in the emission window of eGFP. Black lines represent gating of negative and positive populations. (C) Summary tables reporting the percentage of the gated bacteria from histograms described in panel B. Magenta color represent the total gated bacteria population, red and green describe negative and positive bacteria, respectively. In the last column geometric mean logarithmic values are reported. For each type of cells, three independent experiments were performed with very similar results, as shown here.

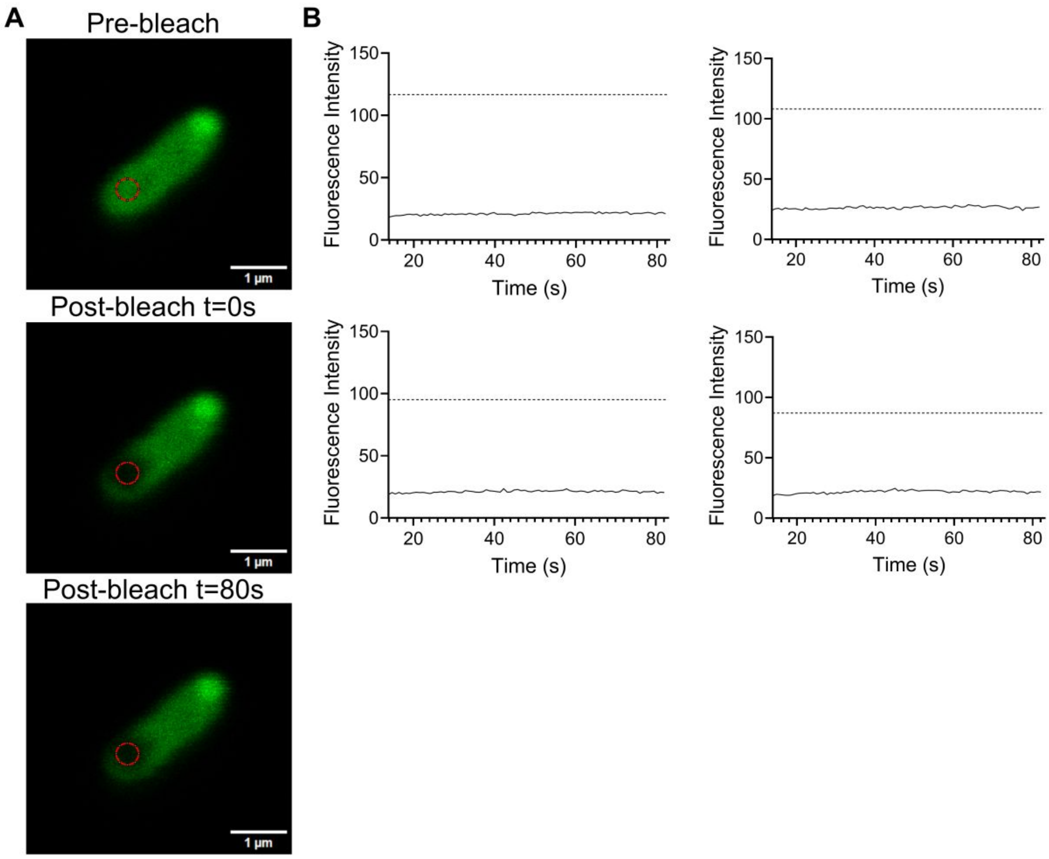

Supplementary Figure S6. FRAP experiments in bacteria expressing wt-trx-eGFP. (A) Representative confocal images of an eGFP-expressing bacterium fixed following the same protocol as used for single-molecule experiments. Top panel: the bacterium before bleaching; middle panel: immediately after the beaching cycle; bottom panel: 80 seconds after bleaching. The bleaching area is highlighted in red. (B) Representative FRAP curves of four different bacteria. Solid lines are the eGFP mean fluorescence intensity within the bleached area over 80 seconds. Dotted lines describe the eGFP mean fluorescence intensity of the pre-bleached area. There is no significant fluorescence recovery after 80 seconds, indicating that protein diffusion is negligible.

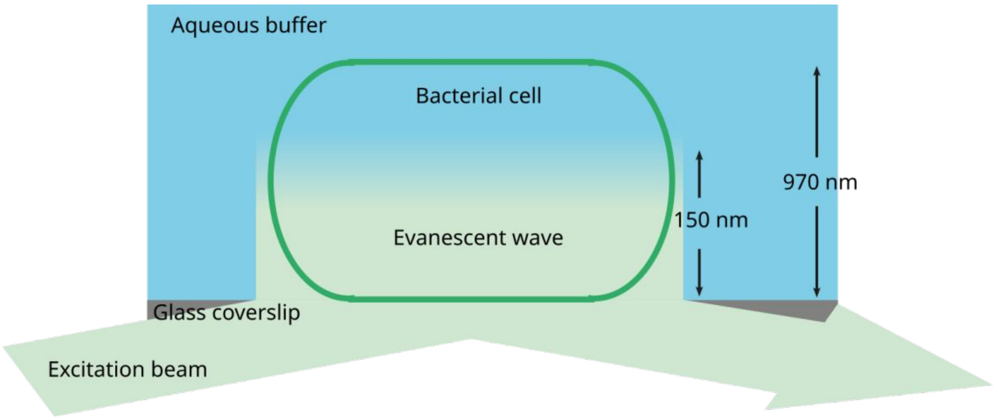

Supplementary Figure S7. Total Internal Reflection Microscopy (TIRF). The excitation beam arrives at the coverslip-aqueous buffer and is totally reflected into the buffer if the incidence angle is larger than the critical angle. In this case, an evanescent wave travels along the interface with an amplitude that falls off exponentially with distance from the interface. Consequently, fluorescent molecules are only efficiently excited in the region closest to the interface (≈150 nm on our microscope). TIRF is, thus, particularly suited for single-molecule fluorescence studies, since background fluorescence is extremely low. The breadth of the bacteria used in this work is ≈970 nm high implying that fluorescent molecules in the bacteria cytoplasm but outside the TIRF excitation volume will not get excited. For this reason, the count of fluorescent molecules within the TIRF volume must be scaled by a factor 6.5 to derive the total molecule count inside the bacterium. Note that an illustrative scheme is displayed here and that the lengths are not shown to scale.

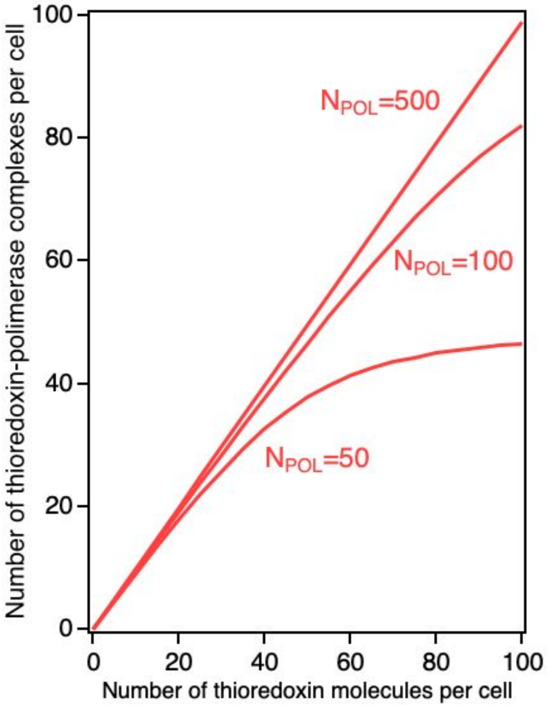

Supplementary Figure S8. Thermodynamic estimation of the number of thioredoxin-viral DNA polymerase complexes within a cell. The interaction between the two proteins to yield the complex, pol+trx↔pol-trx is described by a dissociation constant, K_D=[pol][trx]/[pol-trx], of 5 nanomolar (Hamdan and Richardson, 2009). The concentrations of free (non-bound) polymerase and thioredoxin, [pol] and [trx], that appear in the equation for K_D are related to the corresponding total concentrations through mass balance: $[pol]_T$=[pol]+[pol-trx] and $[trx]_T$=[trx]+[pol-trx]. These two equations can be solved for [pol] and [trx], and subsequent substitution into the expression for K_D yields a second-order equation that can be solved for [pol-trx] as a function of $[pol]_T$ and $[trx]_T$. Finally, concentrations can be converted to numbers of molecules using Avogadro's number and the cell volume. We have used this procedure to calculate profiles of number of complexes versus number of thioredoxin molecules within a single cell for several values of the number of viral DNA polymerase molecules in the cell (N_{POL}). It is likely that, upon infection of a cell, the copy number of viral DNA polymerase reaches very high values. Yet, we have purposedly used low N_{POL} values for these calculations. The profiles obtained show that a dissociation constant of 5 nanomolar predicts that, even with low N_{POL} values, a few tens of thioredoxin molecules in a cell should lead to a few tens of thioredoxin-viral DNA polymerase complexes in the cell.

Ochre codon

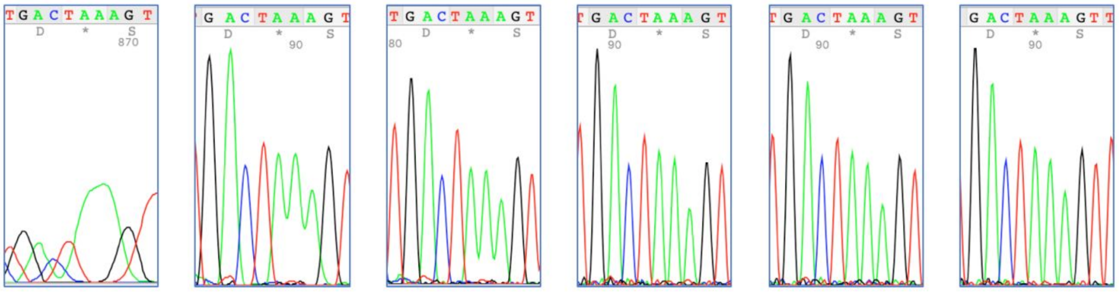

Opal codon

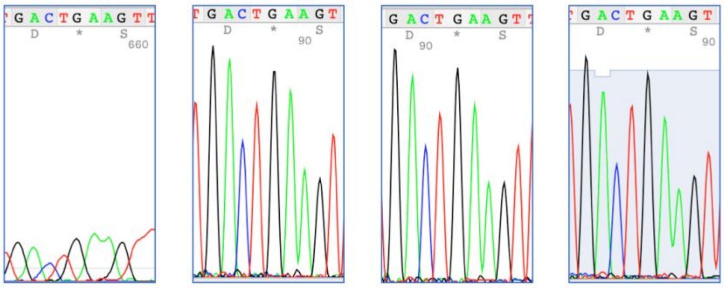

Amber codon

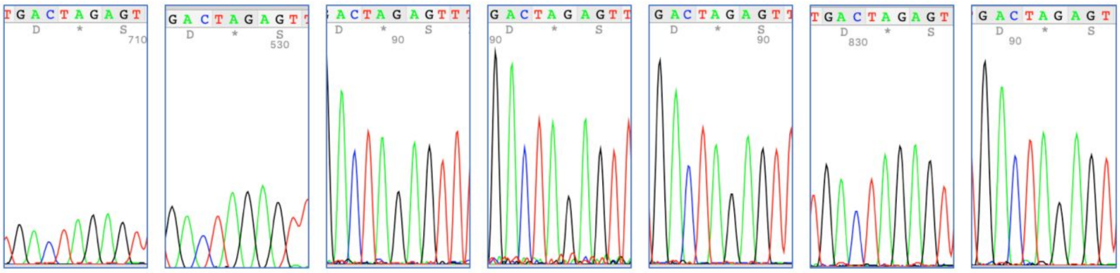

Supplementary Figure S9. Cells transformed with stop-codon bearing constructs were stored as glycerol stocks to be retrieved and grown only immediately before an experiment was planned. We routinely checked that the stop-codon was present trough construct sequencing. The figure shows the results of several independent sequence determinations in the region of the nucleic acid sequence corresponding to position 11 in the amino acid sequence of thioredoxin. In all cases, the targeted stop-codon is identified.

SUPPLEMENTARY DISCUSSIONS

On the factors that may contribute to single-molecule detection overestimating the number of thioredoxin molecules per cell. We detect thioredoxin molecules through the photoconversion of the attached mEos2. Gene expression errors that enable the synthesis of mEos2 might in some cases replace the ochre codon inserted at position 11 with an amino acid residue that brings about destabilizing interactions with its molecular surroundings thus impairing thioredoxin folding. This would obviously contribute to an overestimation of the number of thioredoxin molecules enabling virus amplification because some of the detected trx-mEos2 constructs would not bear a thioredoxin structure competent for interaction with the viral DNA polymerase. mEos2 blinking events (Lee et al., 2012) could be another source of overestimation but given the extremely low number of mEos2 molecules detected, the overestimation due to blinking cannot be but very small.

Another factor that could conceivably contribute to overestimate the number of functional thioredoxin molecules per cell is the possibility that translation is initiated at an alternative start codon after the engineered stop codon. We have examined the mRNA sequence and found that, in the region that encodes thioredoxin, there are only four instances of the common start codons in *E. coli,* (AUG, GUG, UUG) (Gold, 1988). These potential alternative start codons and their corresponding location in the amino acid sequence are: AUG (position 38), GUG (positions 87 and 92) and UUG (position 100). In addition, there is experimental evidence (Hecht et al., 2017) of translation in *E. coli* from start codons other than the most common ones, albeit with very low probability. In any case, initiation of translation requires not only a start codon but also RNA motifs that establish suitable interactions with the ribosome (Reis and Salis, 2020) including a ribosome binding site (RBS) a few bases upstream from the initiation codon (Chen et al., 1994) with the capability to bind to the anti-Shine-Dalgarno (anti-SD) sequence in the ribosome. Our construct does include an RBS sequence a few nucleotides upstream from the original AUG start codon which corresponds to the position 1 in the amino acid sequence or thioredoxin. However, this RBS is many nucleotides upstream of any alternative start codon within the sequence that encodes thioredoxin, which makes it unlikely that translation is initiated at the alternative sites. On the other hand, it appears at least conceivable that some in-frame nucleotide segments display some capability to bind the anti-SD sequence and that these segments are sufficiently close to alternative start codons to enable translation. To explore this possibility, we used the RBS calculator of the "*De Novo* DNA" server (https://www.denovodna.com/#), which takes into account various factors that affect initiation rate (Reis and Salis, 2020). The algorithm predicted initiation from the alternative start codons AUG and UUG corresponding to positions 38 and 100 of the thioredoxin amino acid sequence, but with initiation rates more than 4 orders of magnitude below that for the AUG codon corresponding to position 1 of the amino acid sequence. Clearly, any contribution from initiation at the alternative start codons is minor. Furthermore, any initiation at an alternative start codon would lead to a truncated thioredoxin with an mEos2 protein attached. This expression product would be detected through single-molecule localization, but it would be unlikely to enable virus replication, since the truncated thioredoxin will not yield the 3D-structure that binds the viral DNA-polymerase. Therefore, initiation at the alternative start codons, if

it occurs at all, would lead to an overestimate of the number of thioredoxin molecules and would simply reinforce the central conclusion of this work, namely that virus propagation may be enabled by an exceedingly low number of host-factor molecules.

On the use of an *in vitro* dissociation constant value to evaluate intracellular polymerase-thioredoxin interactions. Some of the illustrative calculations provided in the section "An evolutionary narrative for phage replication at exceedingly low host-factor levels" of the main text are based upon a value of the dissociation equilibrium constant that was determined in *in vitro* assays with purified proteins (Huber et al., 1986). It could be argued that the interaction within a host cell could be effectively weaker than in *in vitro* assays because the viral DNA polymerase would have to compete with many cellular proteins that interact with thioredoxin (Kumar et al., 2004). On the other hand, macromolecular crowding in the intracellular milieu may strongly affect binding equilibria (Rivas and Minton, 2016). One simple possibility is that, since the amount of bulk free solvent in a crowded environment is substantially reduced, the actual thioredoxin and polymerase concentrations are higher than those calculated above using the whole cellular volume. This would contribute to make the polymerase-thioredoxin interaction effectively stronger. It is not clear which one of these two opposing factors, competition with cellular proteins that interact with thioredoxin and increase of effective concentration of the interacting partners, prevails. Yet, the fact that phage replication is enabled by a few tens of host factor molecules supports that assuming an effective nanomolar dissociation constant for calculations on the interaction *in vivo* is, at least, reasonable.

An evolutionary narrative for the tight thioredoxin-polymerase interaction. The interaction between the viral polymerase and the host thioredoxin is very tight, with a dissociation constant in the nanomolar range. However, thioredoxin (Figure 1B) is normally expressed in *E. coli* to copy numbers of about 10000-20000 molecules per cell (Holmgren, 1981; Lunn et al., 1984), implying a normal cellular thioredoxin concentration on the order of tens of micromolar. Then, the obvious question in an evolutionary context is, why would natural selection lead to a very tight nanomolar interaction when a much weaker, even micromolar, interaction should suffice to generate thioredoxin-polymerase complexes and enable virus replication? It could be argued that a very tight binding is required to allow the viral polymerase to compete successfully with the many cellular proteins that interact with thioredoxin (Monici, 2005). However, this interpretation is disfavored by the experimental results reported here which support that polymerase-thioredoxin complexes are formed even with only about ten thioredoxin molecules per cell. On the other hand, a convincing evolutionary interpretation of the tight polymerase-thioredoxin interaction can be easily constructed based on the fact that thioredoxin functions as a processivity factor for the polymerase. This interpretation is provided in the main text, but it is elaborated in some more detail below.

In the absence of thioredoxin, the binding of the viral polymerase to DNA shows limited temporal continuity, in the sense that the polymerase undergoes frequent dissociation/rebinding from/to the DNA. Binding of thioredoxin suppresses this hopping on and off and converts the enzyme in a highly processive polymerase able to synthesize long DNA stretches (Tabor et al., 1987; Etson et al., 2010). This

function of thioredoxin as a processivity factor requires of course that thioredoxin itself does not hop on and off the polymerase. This is so because frequent dissociation of thioredoxin from the polymerase would also bring about frequent dissociation of the polymerase from the DNA (if thioredoxin hops off the polymerase, the polymerase will likely hop off the DNA). Consequently, for thioredoxin to act as a processivity factor, the polymerase-thioredoxin complex must display a sufficiently long residence time. The residence time, that is the average time a complex exists before dissociating, is given by the inverse of the dissociation rate constant, $1/k_{OFF}$ (Copeland, 2016), and reflects, therefore, the kinetic stability of the complex. Furthermore, the equilibrium dissociation constant (K_D) for a 1:1 interaction complex is related to the rate constants for association (k_{ON}) and dissociation (k_{OFF}) through the principle of detailed balance: $K_D=k_{OFF}/k_{ON}$. Therefore, a very low value for the dissociation equilibrium constant, K_D, likely results from natural selection for a very low value of the dissociation rate constant, k_{OFF}, as required to guarantee a long residence time, $1/\ k_{OFF}$, for the thioredoxin-polymerase complex. This interpretation does not rule out, of course, that natural selection for a favorable binding thermodynamics also occurs, but this alone seems unlikely to explain a tight, nanomolar interaction in this case.

Order-of-magnitude estimates of the number of variants bearing phenotypic mutations for proteins involved in the propagation of SARS-CoV2 and influenza viruses. We have proposed and discussed in the main text that viruses may exploit the huge diversity of protein variants present at low level as a result of gene expression errors. We present here some simple order-of-magnitude calculations on the number of protein variants bearing phenotypic mutations that support the plausibility of this claim. While in the main text we deal with the interaction of a viral DNA-polymerase with an essential host factor, we focus here for illustration on virus-host biomolecular interactions that trigger virus entry in a host cell. In general terms, our line of reasoning can be summarized as follows. A single interaction of one viral protein molecule at the surface of one virion with one receptor molecule at the surface of one host cell may trigger the infection of the host cell and the subsequent generation of many new virions. Typically, there are many copies of the crucial viral protein on the surface of one virion and common infective doses comprise very large numbers of virions. There may be, therefore, a huge number of copies of the crucial viral protein in an infective dose and about 20% of those copies will bear phenotypic mutations as a result of translation and transcription errors (Landerer et al., 2024). That is, a wide diversity of variants is generated, and it is statistically plausible that some of these variants bear mutations that enable interaction with the receptor of a new host or antibody evasion. This may enable virus propagation, since infection of even a small number of host cells will lead to the generation of an enormous number of new virions. To illustrate these ideas, we use literature data on SARS-CoV-2 and influenza viruses. Note, however, that the purpose of the calculations described below (and summarized in supplementary Figure 9) is simply to highlight the statistical plausibility of our proposal. The assessment of the extent to which phenotypic mutations contribute to viral adaptation processes must wait future experimental work. In this regard, we conclude this section with some suggestions as to how such the role of phenotypic mutations on viral adaptation could be experimentally addressed.

There are about 300 copies of the spike monomer on the surface of a SARS-CoV-2 virion and infection of a host cell is likely triggered by an interaction of a spike molecule with an ACE2 receptor at the cell surface (Bar-On et al., 2020). Cross-species transmission requires that the virus spike can interact with the ACE2 receptor of the new host. Mutations in the spike linked to cross-species transmission have been identified (Zech et al., 2021; Tan et al., 2022). COVID-19 animal infection models, which may be representative of cross-species transmission, require doses in the 10^4-10^6 range (Brosseau et al., 2022). 10^4-10^6 virions could carry up to several hundred million copies of the spike monomer, out of which about 20% (Landerer et al., 2024), *i.e.* many millions, would be variants with phenotypic mutations (Supplementary Figure 9). It is plausible that, among such a huge number of variants, some harbor phenotypic mutations that enable the interaction of the spike with the ACE2 receptor of the new host. Even a comparatively small number of spike-ACE2 interactions could trigger virus propagation in the new host, since each single interaction would yield an average of 1000 new virions upon infection of a single cell (Bar-On et al., 2020), meaning about 300000 new copies of the spike monomer per cell infected, out of which around 60000 would be again variants with phenotypic mutations (supplementary Figure 10). Of course, the enabling interactions could also be mediated by phenotypic mutations in the receptor, ACE2. Using for illustration a reported value for the copy number of ACE2 in HeLa cells of ~30000 (Kulak et al., 2014) about 6000 ACE2 molecules per host cell would harbor phenotypic mutations. Certainly, virus propagation based on protein variants at low copy number will likely be inefficient but could allow the initial survival of the virus in the new host, thus giving natural selection a chance to act and eventually generate enabling mutations at the genetic level.

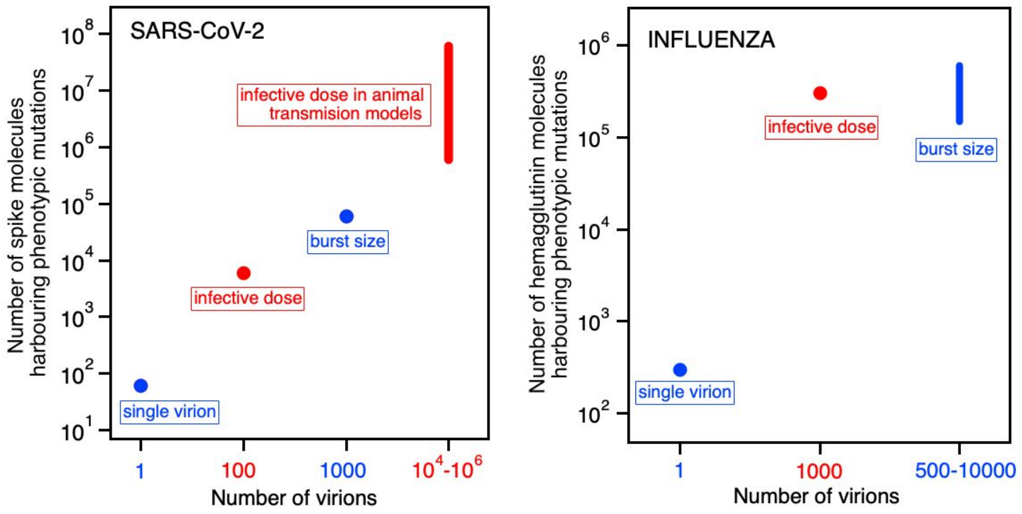

Supplementary Figure 10. Order-of-magnitude estimates of the number of molecules harbouring phenotypic mutations for several instances related to cross-species transmission of SARS-CoV-2 and antibody evasion of influenza virus. Data for SARS-CoV-2 refer to the spike monomer, while data for influenza refer to the hemagglutinin monomer. For these calculations, we have used a recent estimate of 20% for the fraction of proteins bearing phenotypic mutations (Landerer et al., 2024), 300 for the number of spike monomers at the surface of a single SARs-CoV-2 virion (Bar-On et al., 2020) and 1500 for the number hemagglutinin monomers at the surface of a single

influenza virion (Sautto et al., 2018). Estimates of the burst size (number of virions generated in the infection of a single cell) for SARS-CoV-2 and influenza were taken from Bar-On et al. (2020) and Philips and Milo (2015), respectively. Estimates of the minimum infective dose for SARS-CoV-2 and influenza were taken from Karimzaden et al. (2021) and Nikitin et al. (2014), respectively. The infective doses corresponding to animal transmission models of SARS-CoV-2 were estimated as the range of TICD50 or PFU given in Brosseau et al. (2022). In all cases, the calculation performed to obtain the number of molecules harbouring phenotypic mutation is: (number of virions) times (number of molecules at the surface of a single virion) times 0.2.

There are about 1500 copies of the hemagglutinin monomer on the surface of an influenza virion and infection of a host cell is likely triggered by a single interaction of the globular head of the hemagglutinin molecule with sialic acid at the host cell surface (Sautto et al., 2018). Some antibodies bind to the globular head and block the interaction thus preventing infection, but single mutations that evade the antibodies are known (Doud et al., 2018). Some studies have reported a minimal infective dose for influenza virus of about 1000 virions (Nikitin et al., 2014). About 1000 virions could carry more than a million copies of the hemagglutinin monomer, out of which several hundred thousand molecules would be variants harbouring phenotypic mutations (supplementary Figure 9). Several hundred thousand would be a minimum estimate, since in a real-life scenario the infective dose will likely be much higher than the minimum one. In any case, it is plausible that, among such a huge number of variants, some harbour phenotypic mutations that lead to antibody evasion. Even a comparatively small number of such variants could enable virus propagation, since each single interaction with sialic acid could yield a number of new virions in the range from ~500 to ~10000 upon infection of a single cell (Philips and Milo, 1015), meaning millions of new copies of the spike monomer per cell infected, of which several hundred thousand (at least) will be variants with phenotypic mutations (supplementary Figure 10).

It remains now to make some suggestions as to how the role of phenotypic mutations on virus adaptation could be experimentally addressed. One interesting possibility in this context is that phenotypic mutations caused by transcription errors are particularly relevant. It must be noted first that difference between genetic mutations and phenotypic mutations linked to transcription errors applies even to RNA viruses, since the viral RNA-polymerase would generate both genomic RNA to be encapsulated in the virions and mRNAs to be used in protein synthesis. Certainly, the transcription error rate is low as compared with the translation error rate (Drummond and Wilke, 2009). However, transcription errors may have a stronger impact at the protein level because each mRNA molecule is typically translated many times (Traverse and Ochman, 2016). Next-generation sequencing methodologies with the capability to determine mutations present in RNA at very low level are available (Lu et al., 2020). Furthermore, transcription errors are known to be far from completely random (Acevedo et al., 2014; Gout et al., 2017). It would seem then feasible to determine the landscape of transcription errors for viral (and host) proteins and to assess the extent to which the most prevalent phenotypic mutations enable biomolecular interactions crucial for virus propagation.

References for Supplementary Discussion

Acevedo, A.; Brodsky, L; Andino, R. Mutational and fitness landscapes of an RNA virus revealed trough population sequencing. *Nature* **2014,** 505, 686-690.

Bar-On, Y. M.; Flamholz, A.; Phillips, R.; Milo, R. SARS-Cov-2 (COVID-19) by the numbers. *eLife* **2020,** 9, e57309.

Brosseau, L. M.; Escandón, K.; Ulrich, A. K.; Rasmussen, A. L.; Roy. C. J.; Bix, G. J.; Popescu, S. V.; Moore, K. A.; Osterholm, M. T. Severe acute respiratory syndrome coronavirus 2 (SARS-CoV-2) dose, infection, and disease outcomes for coronavirus disease 2019 (COVID 2019): a review. *Clin. Infect. Dis.* **2022,** 75, e1195-e1201.

Chen, H.; Bjerknes, M.; Kumar, R.; Jay. E. Determination of the optimal aligned spacing between the Shine-Dalgarno sequence and the translation initiation codon of *Escherichia coli* mRNAs. *Nucleic Acids Res.* **1994,** 22, 4953-4957.

Copeland, R. A. (2016). The drug-target residence time model: a 10-year retrospective. *Nat. Rev. Drug. Discov.* **2016,** 15, 87-95.

Doud, M. B.; Lee, J. M.; Bloom, J. D. How single mutations affect viral escape from broad and narrow antibodies to H1 influenza hemagglutinin. *Nat Commun.* **2018,** 9, 1386.

Drummond, D. A.; Wilke, C. O. The evolutionary consequences of erroneous protein synthesis. *Nat. Rev. Genet.* **2009,** 10, 715-724.

Etson, C. M.; Hamdan, S. M.; Richardson, C. C.; van Oijen, A. M. Thioredoxin suppresses microscopic hopping of T7 DNA polymerase on duplex DNA. *Proc. Natl. Acad. Sci. USA* **2010,** 107, 1900-1905.

Gold, L. Posttranscriptional regulatory mechanisms in *Escherichia coli. Annu. Rev. Biochem.* **1988,** 57, 199-233.

Gout, J-F.; Li, W.; Fritsch, C.; Li, A.; Haaron, S.; Singh, L.; Hua, D.; Fazelinia, H.; Smith, Z.; Seeholzer, S.; Thomas, K.; Lynch, M.; Vermulst, M. The landscape of transcription errors in eukaryotic cells. *Sci. Adv.* **2017,** 3, e1701484.

Hecht, A.; Glasgow, J.; Jaschke, P. R.; Bawazer, L. A.; Munson, M. S.; Cochran, J. R.; Endy, D.; Salit, M. Measurements of translation initiation from all 64 codons in *E. coli*. *Nucleic Acids. Res.* **2017,** 45, 3615-3626.

Holmgren, A. Thioredoxin: structure and functions. *TIBS* **1981,** 6, 26-29.

Huber, H. E.; Russel, M.; Model, P.; Richardson, C. C. Interactions of mutant thioredoxins of *Escherichia coli* with the gene 5 protein of phage T7. *J. Biol. Chem.* **1986,** 261, 15006-15012.

Karimzadeh, S.; Bhopal, R.; Tien, H. N. Review of infective dose, routes of transmission and outcome of COVID-19 caused by the SARS-COV-2: comparison with other respiratory viruses. *Epidemiol. Infect.* **2021,** 149, e96.

Kulak, N. A.; Pichler, G.; Paron, I.; Nagaraj, N.; Mann, M. Minimal, encapsulated proteomic-sample processing applied to copy-number estimation in eukaryotic cells. *Nat. Methods* **2014,** 11, 319-324.

Kumar, J. K.; Tabor, S.; Richardson, C. C. Proteomic analysis of thioredoxin-targeted proteins in *Escherichia coli. Proc. Natl. Acad. Sci. USA* **2004,** 101, 3759-3764.

Landerer,C., Poehls,J. and Toth-Petroczy,A. Fitness effects of phenotypic mutations at proteome-scale reveal optimality of translation machinery. *Mol. Biol. Evol.* **2024,** 41, msae048.

Lee, S. H.; Shin, J. Y.; Lee. A.; Bustamante, C. (2012) Counting single photoactivatable fluorescent molecules by photoactivated localization microscopy (PALM). *Proc. Natl. Acad. Sci. USA* **2012,** 109, 17436-17441.

Lu, I. N.; Muller, C. P.; He, F. Q. Applying next-generation sequencing to unravel the mutational landscape in viral quasispecies. *Virus Res.***2020,** 283, 197963.

Lunn, C. A.; Kathju, S.; Wallace, B. J.; Kushner, S. R.; Pigiet,V. Amplification and purification of plasmid-encoded thioredoxin from *Escherichia coli* K12. *J. Biol. Chem.* **1984,** 259, 10469-10474.

Monici, M. Cell and tissue autofluorescence research and diagnostic applications. *Biotechnology Annual Review* **2005,** 11, 227–256.

Nikitin, N.; Petrova, E.; Trifonova, E.; Karpova, O. Influenza virus aerosols in the air and their infectiousness. *Adv. Virol.* **2014,** 859090.

Phillips, R.; Milo, R. By the numbers: viral burst size. Posted October-1, **2015**. https://schaechter.asmblog.org/schaechter/2015/10/by-the-numbers-viral-burst-size.html

Reis, A. C.; Salis, H. M. An automated model test system for systematic development and improvement of gene expression models. *ACS Synth. Biol.* **2020,** 9, 3145-3156.

Rivas, G.; Minton, A.P. Macromolecular crowding in vitro, in vivo and in between. *Trends Biochem. Sci.* **2016,** 41, 970-981.

Sautto, G. A.; Kirchenbaum, G. A.; Ross, T. M. Towards a universal influenza vaccine: different approaches for one goal. *Virol. J.* **2018,** 15, 17.

Tabor, S.; Huber, H. E.; Richardson, C. C. *Escherichia coli* thioredoxin confers processivity on the DNA polymerase activity of the gene 5 protein of bacteriophage T7. *J. Biol. Chem.* **1987,** 262,16212-16223.

Tan, C. C. S.; Lam, S. D.; Richard, D.; Owen, C. J.; Bertchtold, D.; Orengo, C.; Nair, M. S.; Kuchipudi, S. V.; Kapur, V.; van Dorp, L.; Balloux, F. Transmission of SARS-CoV-2 from humans to animals and potential host adaptation. *Nat. Commun.* **2022,** 13, 2988.

Traverse, C. C.; Ochman, H. Conserved rates and patterns of transcription errors across bacterial growth states and lifestyles. *Proc. Natl. Acad. Sci. USA* **2016,** 113, 3311-3316.

Zech, F.; Schniertshauer, D.; Jung, C.; Herrman, A.; Cordsmeier, A.; Xie, Q.; Nchioua, R.; Bozzo, C. P.; Volcic, M.; Koepke, L.; Müller, J. A.; Krüger, J.; Heller, S.; Stenger, S.; Hoffman, M.; Pöhlmann, S.; Kleger, A.; Jacob, T.; Conzelmann, K-K.; Ensser, A.; Sparrer, K. M. J.; Kirchoff,F. (2021) Spike residue 403 affects binding of coronavirus spikes to human ACE2. *Nat. Commun.* **2021,** 12, 6855.
